# Linking Cognitive Integrity to Working Memory Dynamics in the Aging Human Brain

**DOI:** 10.1523/JNEUROSCI.1883-23.2024

**Published:** 2024-05-17

**Authors:** Gina Monov, Henrik Stein, Leonie Klock, Juergen Gallinat, Simone Kühn, Tania Lincoln, Katarina Krkovic, Peter R. Murphy, Tobias H. Donner

**Affiliations:** ^1^Section of Computational Cognitive Neuroscience, Department of Neurophysiology & Pathophysiology, University Medical Center Hamburg-Eppendorf, Hamburg 20246, Germany; ^2^Department of Psychiatry, University Medical Center Hamburg-Eppendorf, Hamburg 20246, Germany; ^3^Department of Clinical Psychology and Psychotherapy, Institute of Psychology, University of Hamburg, Hamburg 20146, Germany; ^4^Department of Psychology, Maynooth University, Co. Kildare, Ireland; ^5^Bernstein Center for Computational Neuroscience, Charité Universitätsmedizin, Berlin 10115, Germany

**Keywords:** behavioral modeling, electrophysiology, neural dynamics, neuroimaging, translational neuroscience

## Abstract

Aging is accompanied by a decline of working memory, an important cognitive capacity that involves stimulus-selective neural activity that persists after stimulus presentation. Here, we unraveled working memory dynamics in older human adults (male and female) including those diagnosed with mild cognitive impairment (MCI) using a combination of behavioral modeling, neuropsychological assessment, and MEG recordings of brain activity. Younger adults (male and female) were studied with behavioral modeling only. Participants performed a visuospatial delayed match-to-sample task under systematic manipulation of the delay and distance between sample and test stimuli. Their behavior (match/nonmatch decisions) was fit with a computational model permitting the dissociation of noise in the internal operations underlying the working memory performance from a strategic decision threshold. Task accuracy decreased with delay duration and sample/test proximity. When sample/test distances were small, older adults committed more false alarms than younger adults. The computational model explained the participants’ behavior well. The model parameters reflecting internal noise (not decision threshold) correlated with the precision of stimulus-selective cortical activity measured with MEG during the delay interval. The model uncovered an increase specifically in working memory noise in older compared with younger participants. Furthermore, in the MCI group, but not in the older healthy controls, internal noise correlated with the participants’ clinically assessed cognitive integrity. Our results are consistent with the idea that the stability of working memory contents deteriorates in aging, in a manner that is specifically linked to the overall cognitive integrity of individuals diagnosed with MCI.

## Significance Statement

Several cognitive functions decline during aging, and this process is aggravated in mild cognitive impairment (MCI)—a condition constituting a primary risk factor for developing dementia. One function susceptible to age-related cognitive decline is working memory: the ability to maintain information online for the flexible control of behavior, which entails persistent stimulus-selective neural activity in different regions of the cerebral cortex. We used computational modeling of behavioral and neural recordings to show that the stability of working memory contents is reduced in older human subjects and predicts overall cognitive decline in MCI patients. Our findings provide new mechanistic insight into cognitive aging and MCI and highlight working memory stability as an objective marker of the mechanisms underlying cognitive impairment.

## Introduction

Aging has profound effects on brain function, with important ramifications at the levels of society and individuals (Lindenberger, 2014). While several cognitive capacities tend to decline during aging in a largely correlated fashion (Grady, 2012; Lindenberger, 2014), cognitive aging also exhibits strong interindividual differences (Lindenberger, 2014). These differences may reflect undetected pathology of neural circuitry as well as strategies to cope with performance reductions in specific tasks (Grady, 2012). Developing a mechanistic understanding of these cognitive changes faces several challenges (Grady, 2012). One is to delineate factors that exacerbate the physiological drivers of age-related cognitive decline and incur an increased risk of developing dementia, as manifested, for example, in mild cognitive impairment (MCI; Hedden and Gabrieli, 2004; Grady, 2012; Petersen, 2016). Another challenge is to isolate specific mechanistic markers of aging-related changes in cognitive computation, which predict individuals’ performance on specific cognitive tasks as well as their general cognitive integrity (Grady, 2012).

One capacity affected by age-related cognitive decline is working memory (Park et al., 1996; Hedden and Gabrieli, 2004; Grady, 2012; Lindenberger, 2014). Working memory refers to the ability to maintain information online and put this information to use for cognitive computation and action (Baddeley, 1992). Working memory is an appealing focus for unraveling the physiological basis of age-related cognitive decline for several reasons. First, it is a fundamental building block of cognition (Goldman-Rakic, 1995; Miller et al., 2018), and so individual working memory performance tends to predict performance on a variety of other cognitive tasks (Conway et al., 2002; Oberauer et al., 2008; Salthouse and Pink, 2008; Verhaeghen, 2013). Second, its neural basis, including age-related deteriorations (M. Wang et al., 2011), has been studied extensively in the monkey brain as well as in cortical circuit models (Miller et al., 2018; X-J. Wang, 2021). Third, recent advances in neuroimaging data analysis permit noninvasive tracking of the maintenance of working memory content in the human brain (Christophel et al., 2017; Curtis and Sprague, 2021).

Previous work has culminated in a cohesive framework for linking performance on working memory tasks to computational and neurophysiological mechanisms. The maintenance of information in working memory relies, at least in part, on stimulus-selective neuronal activity that persists after the offset of a to-be-remembered stimulus (Goldman-Rakic, 1995; Miller et al., 2018; X-J. Wang, 2021). Convergent evidence from different approaches in humans and monkeys has identified such content-selective persistent activity in many brain regions including the frontal, parietal, and sensory cortex (Foster et al., 2016; Christophel et al., 2017; Miller et al., 2018; Barbosa et al., 2020, 2021; Curtis and Sprague, 2021; X-J. Wang, 2021). Modeling work shows that such persistent activity can be produced by synaptic reverberation (Compte, 2000; Compte, et al., 2003; X-J. Wang, 2021) in circuits with a balanced interplay between recurrent excitation and inhibition (Shu et al., 2003). Disruptions in this balance deteriorate the stability of the stimulus-selective activity patterns, thus yielding a characteristic decrease of behavioral performance (Murray et al., 2014).

This framework now opens the door for studying the neural bases of age-related changes in cognition at an unprecedented level of mechanistic detail. Abstractions of the above (high-dimensional and biophysically detailed) cortical circuit models can be fit to behavioral data and used to decompose an individual's performance in a working memory task into several latent sources of behavioral variability (Van Den Berg et al., 2012; Kilpatrick et al., 2013; Panichello et al., 2019; Schapiro et al., 2022). This enables a dissociation of the stability of the working memory representation from task strategies that could themselves change with aging.

To illuminate dynamical mechanisms underlying age-related changes in cognition, we aimed to (1) identify computational and neurophysiological signatures of working memory mechanisms and (2) link these signatures to individual cognitive integrity in older adults. To these ends, we combined model-based analyses of behavior during a working memory task with magnetoencephalographic (MEG) recordings. We isolated specific signatures of the stability of working memory representations and the underlying cortical activity during working memory delays.

## Materials and Methods

### Participants and recruitment

We report analyses of two datasets acquired in the context of different studies. Participants of both studies were financially compensated with ten Euros per hour of participation and (for Dataset 2) for expenses incurred due to SARS-CoV-2-antigen testing.

#### Sample for Dataset 1

**Figure 1.**
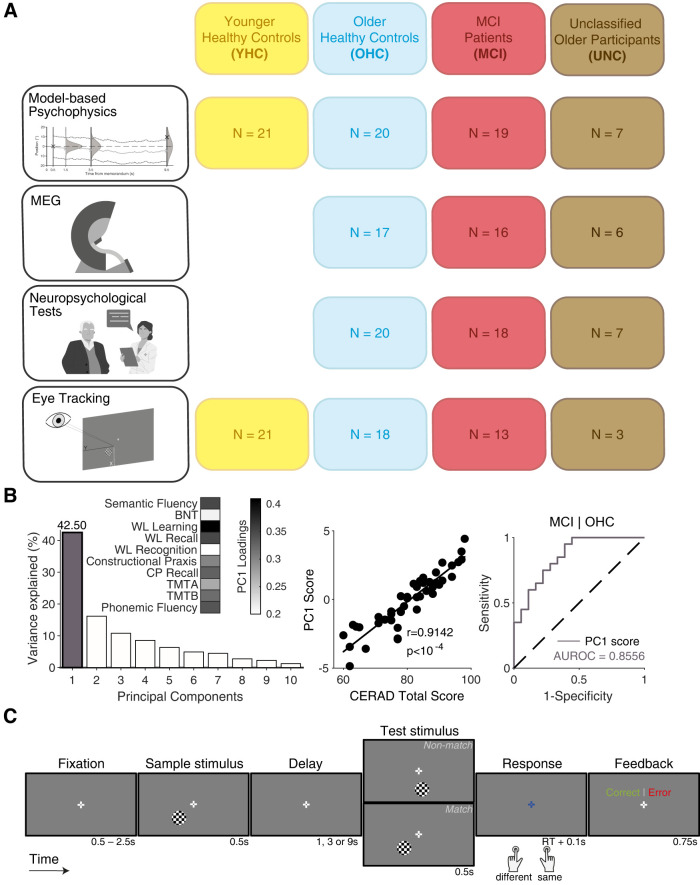
Sample and approach. ***A***, Testing modalities (rows) and definition of participant subgroups (columns). Sample sizes within each modality/subgroup combination define the number of subjects included in the corresponding analyses. ***B***, Left: variance explained by the principal components derived from PCA of the CERAD-Plus neuropsychological test battery data. Inset shows loadings of different tests for the PC1 (gray in plot of variance explained). Middle: correlation of PC1 scores and CERAD total scores across all older adults. Right: ROC curves for diagnostic classification (OHC vs MCI) using PC1 scores (gray). ***C***, Schematic of the delayed match-to-sample working memory task. Appearance of stimuli shown is for illustration purposes only. See Extended Data Figure 1-1*A* and Extended Data Table 1-1 for subgroup-specific performance scores on the CERAD-Plus test battery and Extended Data Figure 1-1*B* for correlation of PC1 scores with MMSE performance. The overall task accuracy for the four groups is shown in Extended Data Figure 1-1*C*. For original appearance of the stimuli, see Extended Data Figure 1-1*D*.

Dataset 1 included behavioral (working memory task), neuropsychological, eye-tracking, and MEG data from three groups of older participants (Fig. 1*A*, Table 1): participants diagnosed clinically with MCI (*N *= 19), age-matched older healthy controls (OHCs; *N* = 20), and unclassified older participants (UNC; *N* = 7). The recruitment of participants and definition of these groups are described in the following.

10.1523/JNEUROSCI.1883-23.2024.f1-1 Figure 1-1 **CERAD-Plus test battery performance scores, validation of composite cognitive score and performance exclusion criterion**. **(A)** Z-scored CERAD-Plus test results (input to principal component analysis) per group (mean ± s.e.m.) show cognitive performance decline in the MCI and UNC groups compared with the OHC group. **(B)** PC1 scores and performance on Mini-Mental State Examination (MMSE) are strongly correlated. **(C)** Single subject working memory task accuracy and kernel density estimates of subgroups show the distributions of task performance measures. The application of task accuracy exclusion criterion at *p*(correct) = 0.6 (dashed black line) leads to the exclusion of three subjects (MCI, N = 2; UNC, N = 1). **(D)** True-sized representation of the stimuli shown to the subjects. *Left to right:* break cue, fixation cross and checkerboard patch, response cue, feedback (correct). Download Figure 1-1, TIF file.

10.1523/JNEUROSCI.1883-23.2024.t1-1 Table 1-1 Group comparison MCI vs. OHC for CERAD-Plus subtest performance. Download Table 1-1, DOCX file.

**Table 1.** Sample description

Measure	YHC (*N* = 21)	OHC (*N* = 20)	MCI (*N* = 19)	UNC (*N* = 7)
Mean age in years (SD)	27.2 (3.8)	69.4 (6.2)	73.7 (6.2)	70.3 (8.2)
% Female	66.7	60	63.2	57.1
% Diagnosis: amnestic MCI | multidomain-amnestic MCI	-	-	63.2 | 36.8	-

YHC, younger healthy controls; OHC, older healthy controls; MCI, mild cognitive impairment; UNC, unclear diagnosis.

Participants for the MCI group were recruited in the outpatient clinic for memory disorders of the Department of Psychiatry and Psychotherapy, University Medical Center Hamburg-Eppendorf (UKE). The study was part of a longitudinal study, in which patients underwent a placebo-controlled intervention (tailored video game). All measurements reported in this paper stem from the first time point (preintervention baseline). Included patients fulfilled diagnostic criteria for MCI (F06.7) according to the International Classification of Diseases, Version 10 (World Health Organization, 2004). Further, the domain-specific subdivisions of amnestic MCI and multidomain-amnestic MCI were both eligible for the study. The distribution of diagnoses within the MCI group is shown in Table 1. Age-matched healthy control participants for the OHC group were recruited using flyers and newspaper/online announcements.

For the assessment of cognitive integrity, the extended version of the well-validated Consortium to Establish a Registry for Alzheimer's Disease (CERAD-Plus) neuropsychological test battery including additional tests for executive functions and cognitive speed (Moms et al., 1989; Mirra et al., 1991; Schmid et al., 2014) was employed. Participants of the OHC group showed no cognitive impairment unexpected for their age, gender, and educational level (population-based *z*-scores greater than −1.5 in each test of the test battery).

Participants originally recruited for the OHC group that produced scores in line with MCI in the neuropsychological assessment could choose to be consulted clinically by a psychiatrist of the outpatient clinic for memory disorders. In case they met the inclusion criteria for the study and the physician deemed it appropriate, participation as an MCI patient in the study was offered, and they were assigned to the MCI group for further analysis (*N* = 1). Those subjects who did not fulfill the criteria for OHC and did not undergo this full diagnostic procedure were not assigned to either the OHC or MCI group and were grouped together as the unclear diagnosis (UNC) group (*N *= 7).

General inclusion criteria were participant's age between 55 and 90 years; ability to consent; place of residence around Hamburg, Germany; and sufficient mobility. Exclusion criteria were relevant psychiatric concomitant diseases (depression, schizophrenia, anxiety disorder, personality disorder), physical illnesses with a relevant influence on mental or motor skills functions (e.g., stroke, heart failure, cerebrovascular diseases, endocrinological disorders, inflammatory diseases of the central nervous system, epilepsy, Parkinson's disease), substance dependence or substance abuse, relevant impairments of the sensory system that make interventions impossible, clinically relevant anemia, nonremovable metal implants or implanted electronic devices, and claustrophobia.

#### Sample for Dataset 2

Dataset 2 consisted of behavioral and eye-tracking data (same working memory task as Dataset 1) from a group of young healthy controls (YHCs, *N* = 21; Fig. 1*A*, Table 1), collected in the context of a separate study at the Department of Clinical Psychology and Psychotherapy at the Universität Hamburg. We used these data as a reference in the current study to identify overall age-dependent changes in working memory performance.

Participants in the YHC group were recruited from the general population of Hamburg through flyers and online announcements and included if they were between 18 and 35 years old and had no pre-existing, diagnosed mental disorder or neurological condition.

#### Informed consent

Participants’ informed (written) consent was obtained in both studies. The study design for both datasets was approved by the ethics committee of the Department of Psychiatry at the UKE (Dataset 1) or the Faculty of Psychology and Human Movement Science at Universität Hamburg (Dataset 2) and conducted in accordance with the Declaration of Helsinki. We excluded subjects from all analyses if their accuracy in the working memory task was below 60% (*N* = 3; Extended Data Fig. 1-1*C*), which is below the accuracy that would be achieved should a participant employ the simple strategy of giving a “different” response on every trial (see below, Working memory task and procedure).

### Experimental design

#### Dataset 1

MEG sessions were conducted in the UKE Department of Neurophysiology and Pathophysiology. For MEG data acquisition, subjects were placed in a comfortable position in a magnetically shielded room, at a viewing distance of 60 cm from the screen on which the stimuli were shown. MEG data were recorded with 275 axial gradiometers (CTF Systems) at a sampling rate of 1,200 Hz. Electrocardiogram (ECG), vertical EOG, and horizontal EOG were measured using bipolar Ag/AgCl electrodes and a ground measured on the wrist. Subjects were asked to minimize their head movements during the measurements, and the three-dimensional head position was recorded via fiducial coils attached to the external auditory canals and the nasion. This permitted online tracking of the head position and guiding of subjects back into their initial position during breaks of the experimental task. Stimuli were backprojected on a transparent screen with a projector (Sanyo PLC-XP51) with 1,920 × 1,080 resolution, at a refresh rate of 60 Hz. Eye movements and pupil size were recorded during task performance with an EyeLink® 1000 long-range mount (SR Research) device at a sampling rate of 1,000 Hz.

Structural T1-weighted MRI scans for individualized source reconstruction of MEG data were collected with a Siemens 3 T MAGNETOM Prisma scanner using a standard 32-channel head coil. The structural images were obtained using a three-dimensional T1-weighted magnetization-prepared gradient-echo sequence (repetition time = 2,500 ms; echo time = 2.12 ms; TI = 1,100 ms; acquisition matrix = 232 × 288 × 19.3; flip angle = 9°; 0.83 × 0.83 × 0.94 mm voxel size).

The neuropsychological assessment of the older subjects was administered by experienced clinical staff in the outpatient center for memory disorders of the UKE Department of Psychiatry and Psychotherapy. The tests of the CERAD-Plus battery were performed in a paper–pencil format and evaluated for diagnostic purposes according to the reference data provided by the Memory Clinic of Basel (Platter, 2018).

#### Dataset 2

Data were collected in a behavioral laboratory under similar conditions as for the MEG recordings in Dataset 1 (task-relevant stimulus parameters were identical with the exception of a shorter intertrial interval (see below, Working memory task and procedure). Here, we used a headrest to ensure a fixed viewing distance of 52 cm from the monitor, and stimuli were presented on a 22″ Dell P2210 monitor with a resolution of 1,680 × 1,050 and a refresh rate of 60 Hz. Eye-tracking (SMI RED500) data were simultaneously acquired during task performance at 250 Hz. ECG data were also recorded but are not reported here.

### Neuropsychological test battery and cognitive integrity score

The neuropsychological diagnostics carried out were based on the validated CERAD-Plus test battery, which consists of 11 individual tests: verbal fluency (semantic, animals; phonemic, *S*-words), modified Boston Naming Test (BNT), Mini-Mental State Examination (MMSE), word list (learning, recall, and recognition), constructional praxis, constructional praxis recall, and Parts A and B of the trail making test (TMT). As is common in the field, performance on the word list recognition test was quantified in terms of the discriminability score introduced in Mohs et al. (1986):

(1) $$\mathrm{Discriminability}=\left(\;1-\frac{\left(10-H)+(10-CR\right)}{20}\right)\times 100,\;\;$$

where *H* was the hit rate and CR was the rate of correct rejects.

We used principal component analysis (PCA) to combine ten test scores into a single summary measure of cognitive integrity, whereby the MMSE was excluded as scores on this test already reflected a mixture of assessments of several cognitive domains. The strong correlation between MMSE scores and our cognitive summary score (*r *= 0.65; *p *< 10^−4^) further validated our approach (Extended Data Fig. 1-1*B*). To compute the summary measure of cognitive integrity, the individual scores on each test were *z*-transformed across subjects (Extended Data Fig. 1-1*A*). Unlike the other tests, high scores of both TMTs (Parts A and B) reflect poor performance. To ensure that positive values reflected higher performance for all tests, the signs of the *z*-scores for these two tests were flipped. We then computed the (across-subject) covariance matrix between the *z*-scored test scores (dimensionality, 10 × 10) and used MATLAB's (MathWorks®) singular value decomposition algorithm to compute the corresponding ten principal components and their associated eigenvalues. One subject (MCI) was excluded from this analysis because they did not complete all tests in the battery.

For comparison with previous neuropsychological work, we also computed a composite CERAD total score proposed by Chandler et al. (2005) as the sum of the following six test scores: semantic fluency (max. 24 points), BNT, word list learning, word list recall, word list recognition (truepositives−falsepositives) and constructional praxis. This CERAD total score was closely correlated with the eigenvalue of the first principal component (PC1) derived via the PCA procedure described above (Fig. 1*B*, middle). In this study, we used the PC1 as the summary measure of individual cognitive integrity. We took this approach because it made use of information from all tests of the extended test battery version, which has been shown to be diagnostically beneficial (Schmid et al., 2014), and because in doing so, tests that do not contribute to the CERAD total score, but that showed group differences between OHC and MCI in our study, could be included in the composite score for cognitive integrity (Extended Data Table 1-1). The PC1 explained a large fraction of variance (>40%) in the neuropsychological data (see Results).

### Working memory task and procedure

The visuospatial delayed match-to-sample working memory task (Fig. 1*C*; Extended Data Fig. 1-1*D*) was programmed in MATLAB using Psychtoolbox-3 (Brainard, 1997). The task was to decide whether a sample stimulus and a test stimulus separated by a variable delay occurred in the same or different locations. Each trial began with presentation of a central white fixation cross (arm length, 0.8° of visual angle, d.v.a.; arm thickness, 0.2 d.v.a.) that was present for the entire trial. After a variable baseline interval (uniform distribution with range 1–2.5 s for Dataset 1, 0.5–2.0 s for Dataset 2), the sample stimulus was presented for 0.5 s, followed by the delay (1, 3, or 9 s, equiprobable) and then the test stimulus (0.5 s). Sample and test stimuli were circular checkerboard patches (diameter, 2.8 d.v.a.; spatial frequency, one cycle per d.v.a.), appearing in the lower visual hemifield at a fixed eccentricity of 6 d.v.a. The sample could be presented at any of 12 equiprobable locations, ranging from ∼13.85 to ∼166.15° of polar angle (fixed spacing ≈ 13.85°), while the most extreme samples could still be flanked by a “near nonmatch” test stimulus on both sides (amounting to 14 possible test stimulus locations, ranging from 0 to 180°). Herein, 0° refers to the left part of the horizontal meridian. The test occurred at either the same location as the sample or at a different location (see below). Upon offset of the test stimulus, the fixation cross changed color from white to light blue, which prompted subjects to report their decision via right- or left-handed button press for “same” or “different” judgments, respectively. This response was soon (0.1 s) followed by visual feedback about its accuracy (“correct” in green font; “error” in red font; font size 36, presented 1.0 d.v.a. above fixation for 0.75 s). Each trial was followed by a fixed interval (3 s for Dataset 1, 2 s for Dataset 2) during which participants were instructed to blink if needed, and this was followed by the baseline period of the following trial.

The task was designed to consist of three trial categories, each with a desired frequency of occurrence within a block of 63 trials: “match trials” (sample and test at identical positions, 33% of trials), “near nonmatch trials” (smallest possible sample-test distance of 13.85°; 33% of trials), and “far nonmatch trials” (sample-test distance randomly chosen from the remaining possible sample-test distances, which could be between 27.7 and ∼166.15° depending on the sample location; 34% of trials). Trials were presented in blocks of 63 trials each, within which the different delay durations and sample-test distances were randomly interleaved under the abovementioned constraint. Subjects received feedback about their average performance at the end of each block. They were instructed to fixate the central cross and minimize blinking during the trial.

Before starting the experimental task, all subjects underwent training to familiarize them with the task. This consisted of a general instruction of the task rules with the help of a slide presentation (administered outside the MEG chamber for Dataset 1 and in the testing room for Dataset 2) and practice with various aspects of the task (after the subject had been placed in the MEG for Dataset 1 and in the testing room for Dataset 2). The first stage of the practice required the participant to fixate the fixation cross while checkerboard stimuli identical to those of the main experiment were presented, including feedback if and when the participant broke fixation. Next, four nonconsecutive example trials covering match, far nonmatch, and near nonmatch trials and varying delay durations were performed. In case of an incorrect response on any of these trials, a text with the correct solution was displayed and the trial was repeated. Finally, six consecutive trials were performed with identical timing and intertrial intervals as in the main experiment. Once training was complete, each subject then performed several blocks of the main experiment (concurrent to MEG measurement for Dataset 1).

We aimed for three task blocks per subject in Dataset 1 and at least two blocks per subject in Dataset 2. Data collection had to be terminated early in some subjects, due to lack of alertness or willingness to continue, or end of the scheduled testing session. We obtained the complete set of three trial blocks for the following fractions of participants per group: MCI, 14/19; OHC, 13/20; UNC, 3/7; and YHC, 4/21. Since the differences in trial counts for different individuals/groups studied here only affect the precision of the parameter estimates (behavioral model parameters or MEG measures), but did not bias them in a particular direction, they also did not bias the group comparisons or across-subject correlations reported in this paper. Furthermore, we included these trial counts as nuisance regressors in regression models (Fig. 7).

### Analysis of working memory-guided behavior

Trials were excluded from analysis contingent on the following criteria: a task-irrelevant button (two of four available buttons on the response pad) was pressed, response time was ≤0.2 s, or it exceeded the subject's mean response time by four standard deviations. Further, the entire first block of one MCI participant was excluded from analysis due to poor comprehension of task instructions (accuracy <50% correct for this block). Accordingly, an average (SD) of 157 (31) trials per subject were submitted for analysis (range across subjects, 59–189 trials).

**Figure 2.**
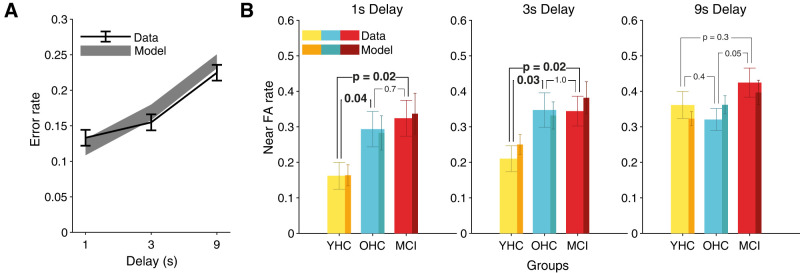
Behavioral results. ***A***, Error rate (black, mean ± SEM) as a function of delay duration across all subjects (*N* = 67) and corresponding model predictions (gray, mean ± SEM). ***B***, False alarm rate on near nonmatch trials by delay durations (lighter shades, mean ± SEM) and corresponding model predictions (darker shades, mean ± SEM) for YHC (*N* = 21; yellow), OHC (*N* = 20; blue), and MCI (*N* = 19; red) subgroups. *P*-values refer to results of nonparametric, between-subject permutation tests. See Extended Data Figure 2-1 for error rates and model predictions plotted separately for all participant groups, trial types, and delay durations.

The mean response accuracy was computed as the proportion of correct responses (i.e., “same” response on match trials and “different” response on nonmatch trials). For several analyses (Fig. 2*B*; Extended Data Fig. 2-1), error rates were analyzed separately for the three trial categories described above.

We also quantified the signal-detection theoretic (Green and Swets, 1966) measures of sensitivity $\left(d^{\prime }\right)$ and criterion (c) from the fractions of hits (“same” responses on match trials, denoted as H) and false alarms (“same” responses on nonmatch trials, denoted as FA), whereby the latter fraction was first computed separately for the near and far nonmatch trials and then averaged.

Sensitivity $d^{\prime }$ and criterion c were then computed as follows:

(2) $$d^{\prime }=z(H)-z(\mathrm{FA}),$$

(3) c=z(H)+z(FA).

### Computational modeling of working memory-guided behavior

#### General approach

Our model of working memory task behavior consisted of two computational elements, both of which accounted for a fraction of behavioral imprecision: (1) a point-estimate memory representation that diffused over time, leading to an increase in error as a function of delay (Panichello et al., 2019), and (2) a decision transformation of that representation into a categorical behavioral report (Engel and Wang, 2011; Murray et al., 2014).

The dynamics of the memory representation were modeled as a Wiener diffusion process where the standard deviation of the across-trial distribution of memory representations at generic time *t* during the memory delay was captured by *σ_t_* and determined by the memory noise parameter *σ*_mem_ as follows:

(4) $$\sigma_{t}\;=\;\sqrt{t\sigma_{\mathrm{mem}}^{2}}.$$

The across-trial variance of this memory representation thus increased monotonically as a function of time (Eq. 4). In practice, we estimated *σ_T_*, the across-trial memory representation distribution at the end of a given delay duration *T* = {1, 3, 9} s. This representation was then transformed into a probability distribution over the task-relevant decision variable x, the absolute distance between the memory representation and test stimulus presented at the end of the delay duration T. We modeled the across-trial distribution of the decision variable as a normal distribution with a mean equal to the true sample-test distance (Δ) and folded around zero (corresponding to test location) to reflect the absolute deviation of the internal memory representation from the test stimulus as follows:

(5) $$\mathrm{pdf}(x|\Delta ,\sigma_{T})=\frac{1}{\sqrt{2\pi \sigma_{T}^{2}}}\times dx\times \exp\left(-\frac{{\left(x\text{–}\Delta \right)}^{2}}{2\sigma_{T}^{2}}\right)+\;\frac{1}{\sqrt{2\pi \sigma_{T}^{2}}}\times dx\times \exp\left(-\frac{{\left(x+\Delta \right)}^{2}}{2\sigma_{T}^{2}}\right).$$

Here, the two summed terms on the right-hand side of the equation correspond to the probability densities from the original memory representation distribution, taking an equal distance (*x*) left and right of the test stimulus location. We evaluated this function numerically for 0≤x≤360 (with the high upper bound allowing for possible cases of very high memory noise) and at a resolution dx=0.05. We modeled the decision function (DF) for a given value of x as a logistic function:

(6) $$\mathrm{DF}(x|\theta ,\delta ,\sigma_{\mathrm{dec}})\;=\;\theta +\frac{1\text{–}(2\times \theta )}{1+\;e^{-(x\text{–}\delta )/\sigma_{\mathrm{dec}}}\;\;}\;,$$

where *δ* was the inflection point of the function [DF (*δ*) = 0.5] and corresponds to a “soft” (i.e., nondeterministic) threshold for translating x into a same (DF << 0.5) or different (DF >> 0.5) choice; *σ*_dec_ was a decision noise parameter that governed the slope of DF; and *θ* was the probability of a time-independent lapse that determined the function's two asymptotes (*θ* and 1-*θ*, respectively), assumed to be symmetric for simplicity. The probability of a “different” response as a function of delay duration T and sample-test distance Δ was then computed by integrating (i.e., summing) over all x:

(7) p(different|T,Δ)=∑DF(x)×pdf(x|Δ,T).

**Figure 3.**
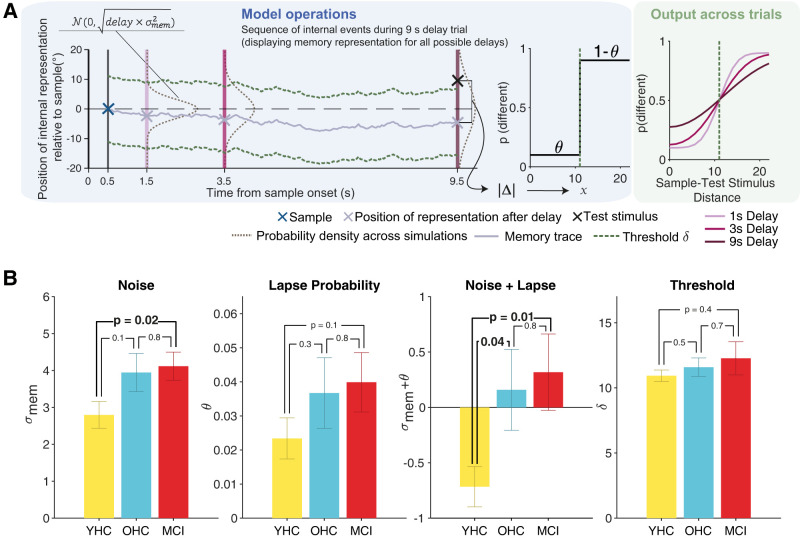
Behavioral modeling results. ***A***, Left: model schematic with one exemplary memory trace (solid light gray line) diffusing in space over time from sample (blue cross) offset (solid dark gray line). The diffusion causes the across-trial probability density of the memory trace to broaden over time (dashed brown lines at delay durations of 1, 3, and 9 s). At the end of the delay (3 purple lines corresponding to the 3 delay durations used here), there is a readout of the memory representation (gray crosses). In the example shown here, a “nonmatch” test stimulus (black cross) is presented after 9 s of delay (dark purple). The dashed green line represents the decision threshold, which produces a “correct” response in this example as the test stimulus lies outside the threshold. Middle: DF for translating memory representation-test stimulus distance into probability of a “different” response, with symmetric asymptotes defined by the value of the time-independent lapse probability (*θ*). Right: model-derived choice probabilities for reporting “different” as a function of sample-test stimulus distance for each delay duration (shades of purple). ***B***, Fitted model parameters for each subject group (mean ± SEM) and *p*-values of associated between-subject nonparametric permutation tests. See Extended Data Figure 3-1 for model validation and comparison and Extended Data Figure 3-2 for analyses on interdependence of memory noise and threshold parameters.

Note that lapses could be sensory, motor, decisional, or mnemonic in origin. Also note that, if DF took the form of a step function (i.e., infinite slope, *σ*_dec _= 0), then the location of the step was equivalent to a deterministic threshold applied to the decision variable (Fig. 3*A*, middle).

In some model variants, we also allowed for the possibility of time-dependent memory lapses. The time-dependent memory lapse probability was modeled as a hazard function in which the likelihood of a memory lapse having occurred by time *t* during the delay $\left(\theta_{\mathrm{mem},t}\right)$ accumulated over time:

(8) $$\theta_{\mathrm{mem},t}\;=\;1\;-\;\;e^{-\lambda t},$$

where λ was the hazard rate.

The overall choice probability was then computed as a mixture model as follows:

(9) $$p(\mathrm{different}|T,\Delta )=\theta_{\mathrm{mem},T}\times 0.5+(1-\theta_{\mathrm{mem},T})\times \sum \mathrm{DF}(x)\times \mathrm{pdf}(x|T,\Delta ).$$

We fit six different variants of this general model to participants’ choices (Table 2).

**Table 2.** Free parameters in fitted model variants

Model #	Memory noise (*σ*_mem)_	Threshold (*δ*)	Lapse (*θ*)	Memory lapse rate (*λ*)	Decision noise (*σ*_dec_)	# of free parameters
1	X	X				2
2	X	X	X			3
3	X	X		X		3
4	X	X			X	3
5	X	X	X	X		4
6	X	X	X	X	X	5

The most complex model variant (Model 6) allowed all five above-described parameters to vary: memory noise *σ*_mem_, decision noise *σ*_dec_, decision threshold *δ*, fixed lapse probability *θ*, and hazard rate of memory lapses *λ*. The simplest model variant (Model 1) fit only *σ*_mem_ and *δ* as free parameters. All variants of intermediate complexity fit *σ*_mem_ and *δ* as additional free parameters. In variants that fit only a subset of the five parameters described above, all other parameters were set to zero. Model variants not including time-independent (*θ*) and time-dependent (*λ*) lapses resulted in asymptotes of the DF equal to 0 and 1. Model variants in which decision noise (*σ*_dec_) was set to zero resulted in a DF that took the form of a step function (Fig. 3*A*, middle) as opposed to a smooth sigmoid.

#### Parameter estimation

The objective function to be minimized during model fitting was defined as the cross-entropy across trials trl between the participants’ responses and model predictions for the likelihood of a “different” response:

(10) $$\;\mathrm{CE}=-\underset{\mathrm{trl}}{\sum }\;(1-pd_{\mathrm{trl}})\log(1-\;{\hat{\;pd}}_{\mathrm{trl}})+pd_{\mathrm{trl}}\log({\hat{\;pd}}_{\mathrm{trl}}),\;$$

where $pd_{\mathrm{trl}}$ refers to the participant's response and ${\hat{\;pd}}_{\mathrm{trl}}$ to the model's prediction. Best-fitting parameters for a particular model variant were found by minimizing this objective function using the particle swarm optimization algorithm (100 particles with wide parameter bounds and initialized at pseudorandom locations: max. of 1,000 iterations) using a toolbox designed for implementation in MATLAB (Birge, 2003).

#### Model selection: parameter recovery and model comparison

Among the six candidate fitted models (Table 2), we selected a single variant through a combination of parameter recovery and formal model comparison, and the parameter estimates of this variant were then used for all analyses reported in “Results.” To evaluate the recoverability of parameters from the different candidate model variants, we simulated behavioral datasets (*N* = 100, each consisting of 189 trials) for each variant where each parameter was set to a representative value across all subjects from fits of the least complex model that included that parameter (*σ*_mem _= 4.2856; *σ*_dec _= 3.0802; *δ* = 11.1370; *θ*_dec _= 0.0203; *λ* = 0.0049; Extended Data Fig. 3-1*A*). We evaluated the recovery of *σ*_mem_ and *δ* (key parameters present in all model variants) by means of the width of the distribution of the fitted parameters across all 100 simulated datasets. This procedure showed that the recoverability of *σ*_mem_ and *δ* was compromised in models that included *σ*_dec_ as a free parameter (Extended Data Fig. 3-1*B*), excluding those two models from further consideration.

Next, the goodness of fit of the remaining four model variants was compared by means of Bayes’ information criterion (BIC) scores as follows:

(11) $$\mathrm{BIC}=2\;\times CE+n_{\mathrm{free}\;\mathrm{parameters}}\times \log(n_{\mathrm{trials}}).$$

Model 2, which included *σ*_mem_, *δ*, and *θ* as free parameters, was the model with the lowest mean BIC score when pooling all subjects across groups (Extended Data Fig. 3-1*C*). Taken together, parameter recovery analyses and model comparison motivated our selection of Model 2 (Table 2) for all further analyses of the parameter estimates (link to overt behavior, cognitive integrity, and MEG). We acknowledge that it remains possible that time-dependent memory lapses and/or decision noise affected participants’ behavior. However, our parameter recovery analysis suggests that the current experimental manipulations are not adequate for identifying the contributions of these additional parameters.

### MEG data analysis

MEG data were analyzed with a combination of customized scripts (see associated code) and the following toolboxes: FieldTrip (Oostenveld et al., 2011) for MATLAB and MNE (Gramfort et al., 2013) and pymeg for Python (https://github.com/DonnerLab/pymeg), as established in the previous work of our laboratory (Wilming et al., 2020; Murphy et al., 2021).

#### Preprocessing

Preprocessing of the MEG data proceeded according to a standardized pipeline developed in our laboratory (https://github.com/DonnerLab/meg-preproc). This broadly involved initial artifact detection and removal using both independent component analysis (ICA) and non-ICA methods, trial segmentation, and reconstruction of the signal with respect to its cortical source.

Continuous MEG time series for each task block were first resampled to 400 Hz, and the line noise was removed by bandstop filtering ∼50, 100, and 150 Hz. Time points at which any of the following MEG artifacts occurred were then identified: head movements (translation of any fiducial coil >6 mm from the first data point in that block), muscle artifacts (*z *> 20 after applying a 110–140 Hz Butterworth filter and *z*-scoring), sensor jumps (detected by Grubb's outlier test for intercepts of lines fitted to log-power spectra computed on 7 s data segments, with 20% overlap between successive segments), and other noise sources (usually due to cars passing the MEG laboratory, identified as any 2 s data segment in which any sensor had data range >20 pT). The timings of blinks (EyeLink algorithm) and saccades (gaze changes >1.5 d.v.a.) were identified using the eye-tracking data if these data were deemed of sufficient quality after visual inspection, or through outliers in the vertical EOG (*z*-score > 2). Heartbeat timings were identified by applying FieldTrip's *ft_artifact_ecg* algorithm to the ECG data.

Having detected the artifacts described above, we then high-pass filtered the continuous MEG data at 1 Hz, discarded any time points containing head movement, muscle, jump, or car/other artifacts, concatenated the cleaned time series across blocks for a given participant, and subjected the resulting concatenated data to ICA (infomax algorithm). Component time series were then segmented from −1 to 2 s around identified blinks and saccades (−0.3–0.3 s around identified heart beats); components were ranked by their coherence with EOG (ECG) data equivalently segmented around blinks/saccades (heart beats); the temporal trajectories, spectral properties, and spatial topographies of the 25 components with the highest coherences were visually inspected; and the component numbers of those judged to capture eye or cardiac artifacts were noted. In a final set of steps, the ICA weights were backprojected onto the downsampled, bandstop-filtered continuous MEG data now subjected to a high-pass filter of 0.1 Hz; those components capturing eye and cardiac artifacts were removed from the data; and the data were epoched into trial intervals from 0.6 s before sample onset to 0.3 s after test stimulus onset. In addition to those excluded due to the response time and accuracy criteria described above for behavioral analyses, trials with any time point containing previously identified head movement, muscle, jump, or car/other artifacts were excluded from all MEG analyses.

Subjects who despite careful recruitment were judged through visual inspection to be subject to persistent artifacts in their MEG data (presumably from the presence of metallic materials, used for example in dental work) were preprocessed without discarding these time points, and the data quality was re-evaluated after the source reconstruction procedure (*N* = 7). Subjects whose data on visual inspection after source reconstruction still displayed slow fluctuations indicating metal artifacts were excluded from further MEG data analysis altogether (*N* = 3).

Our MEG analyses focused primarily on data around sample presentation and the first second of the delay. If trials with a delay duration above 1 s did not contain artifacts during the first second of the delay, these were preserved for the analysis together with the 1 s delay trials to increase the number of trials available for analysis and computation of the data covariance matrix (see below, Spectral analysis and source reconstruction). If a cleaned dataset for an individual subject consisted of fewer than 80 trials after preprocessing, then that subject was excluded from all MEG analyses (*N* = 4). In total, this procedure resulted in the exclusion of 7 of the 46 subjects who contributed to the behavioral results, leaving 39 subjects for MEG analysis in total (16 for MCI, 17 for OHC, and 6 for UNC).

#### Spectral analysis and source reconstruction

We first subjected the trial-averaged (phase-locked) response of each sensor from the single-trial time courses, in order to isolate activity components that are nonphase locked to stimulus onset. The latter are generated by recurrent synaptic interactions that are also involved in the generation of persistent cortical activity (Donner and Siegel, 2011; Miller et al., 2018). Time-frequency representations (TFRs) of complex-valued Fourier coefficients (phase and amplitude information) for individual trials were calculated using a sliding window Fourier transform. We used Hanning tapers for the frequency range 1–35 Hz (window length, 0.4 s; time steps, 0.05 s; frequency steps, 1 Hz; frequency smoothing, ±2.5 Hz) and the multitaper method with discrete proloid slepian tapers (window length, 0.25 s; time steps, 0.05 s; frequency steps, 4 Hz; frequency smoothing, ±6 Hz) for the frequency range 36–120 Hz.

For the source reconstruction, we used (mostly individual subject; see below) structural MRI scans to generate three-layered head models in FieldTrip, which were in turn used to compute the forward solution (leadfield) for each source point. The cortical surface was reconstructed using FreeSurfer (Dale et al., 1999; Fischl et al., 1999) and aligned to established anatomical atlases (see below, Definition of ROIs and ROI groups). For subjects with artifactual (*N* = 6) or no (*N* = 4) MRI scans, a template average surface provided by FreeSurfer (“fsaverage”) was used instead. We used linearly constrained minimum variance (LCMV) beamforming to project the sensor-level Fourier coefficients into source space, specifically onto 4,096 vertices per hemisphere located on the cortical surface (recursively subdivided octahedron). We computed LCMV filters with MNE using a covariance matrix of the cleaned, epoched single-trial (broadband) data to constrain a forward model. This covariance matrix was computed using the data from all trials irrespective of delay duration between 0.25 s before and 1.5 s after sample onset. At each vertex, the source orientation was selected based on the maximum output source power determined through singular value decomposition. To overcome random sign flips of the beamformer results, the polarity of the time series of adjacent vertices was aligned. Then the complex-valued Fourier coefficients of each vertex were computed by application of the corresponding spatial filter and transformed into power by taking the absolute value and squaring.

The source-level power estimates were averaged across all vertices within each region of interest (ROI; see below, Definition of ROIs and ROI groups) and normalized with respect to the mean baseline spectrum using the decibel transform. The baseline spectrum was computed by averaging power estimates across the interval −0.4 to −0.2 s from the sample onset and then across all trials. Because we had no a priori hypotheses about hemispheric lateralization effects and the anatomical atlas we used is symmetric, the power modulation values were further pooled across the left- and right-hemispheric parts of each ROI. Power modulations of the ROI groups were evaluated as the mean trial-averaged power values of the ROIs within each ROI group (Table 3).

**Table 3.** Definition of groups of ROIs for time courses of delay activity

Group	ROI	Reference
V1	V1	Glasser et al. (2016)
Early visual	V2, V3, V4	
Dorsal visual	V6, V3A, V7, IPS1, V3B, V6A	
MT+ and neighbors	MST, LO1, LO2, MT, V4t, FST, LO3, V3CD, PH	
Ventral visual	V8, FFC, PIT, VMV1, VMV2, VMV3, VVC	
PMd	6a, 6d	
Eye fields	FEF, PEF	
PMv	6v, 6r	
M1	M1 (Hand)	de Gee et al. (2017)

V1, primary visual cortex; MT+, middle temporal area and medial superior temporal area; PMd, dorsal premotor cortex; PMv, ventral premotor cortex; M1, primary motor cortex.

#### Decoding of sample spatial location from MEG data

We trained multivariate decoders to predict the angular position of the sample stimulus from the spatiospectral power modulation patterns in each cortical area during the trial. Decoding was performed using ridge regression through scikit-learn for Python (Pedregosa et al., 2011), separately for each subject and time point. We normalized the power values of all vertices per “brain region” (from both hemispheres) and 31 frequency bins (range, 5–35 Hz) by *z*-scoring across trials. This frequency range was chosen because we observed sustained power modulations in this range throughout delay intervals (Fig. 5*A*; Extended Data Fig. 5-1*A*). In separate versions of this analysis, “brain region” referred to individual ROIs from the anatomical atlas or ROI groups (Table 3), across which all vertices were pooled. To reduce the dimensionality of the data, PCA was performed on the training data, and we only used the components accounting for top 80% of the variance in the data for the decoding. This cutoff was chosen to avoid overfitting given the relatively low number of trials and resulted in 39.49 ± 8.5 (mean ± SD) components averaged across all time points for example area V1 (Fig. 6*A*). The resulting components were used as decoding features. The decoder was fit using 10-fold cross-validation and an L2 penalty of *α* = 1. Decoding precision was evaluated by Pearson’s correlation coefficient between the predicted sample angle and its actual angle. To additionally assess whether a more coarse-grained decoding approach would boost the sensitivity of the analysis, we also decoded the visual hemifield that the sample stimulus appeared in using cross-validated logistic regression (L2 penalty, *C* = 1). Here, we evaluated the decoder performance using the area under the receiver operating curve (Extended Data Fig. 6-1*A*).

To assess the stability of the working memory code during the delay period, across the entire cortex as well as specifically in the dorsal visual cortex, we computed the cross-temporal decoding precision for the exact sample location by training the decoder at each time point and evaluating its performance at all time points (also known as temporal generalization analysis; King and Dehaene, 2014). The resulting temporal generalization matrix A consists of elements that represent the decoding precisions for each train and test time (x,y).

As a measure of the level of temporal generalization in individual subjects, we determined the sum of all off-diagonal elements during the delay (1s;x,y=[0.5s,1.5s]):

(12) $$\sum A_{x,y}(\mathrm{Test}\;\mathrm{time}\neq \mathrm{Train}\;\mathrm{time}),$$

We defined another measure of temporal generalization with wider shifts between train and test time, removing potential above-chance generalization due solely to the smoothing effect of the window for spectral analysis (0.4 s):

(13) $$\sum A_{x,y}(|\mathrm{Test}\;\mathrm{time}-\mathrm{Train}\;\mathrm{time}|\geq 0.5\;\;s).$$

Finally, to complement our assessment of the above-chance temporal generalization, we also sought to test the evidence for a dynamic code during working memory maintenance. Following previous work (Myers et al., 2015; Wolff et al., 2017), we tested the difference in decoding precision between each cross-time element $\left(A_{x,y}\right)$ with their corresponding within-time elements $\left(A_{x,x\;}\mathrm{and}\;A_{y,y}\right)$ using two-sided nonparametric permutation tests. The presence of a significant difference at time x,y and both within-time elements is considered an indication of dynamic coding. To account for multiple comparisons, these individual sets of *p*-values were false discovery rate (FDR) corrected (Extended Data Fig. 8-1).

#### Predicting individual cognition from MEG markers of cortical delay activity

In order to relate markers of cortical delay activity to computational model-based or neuropsychological test scores, we focused on four markers derived from the spectral power modulations and evaluated across the first 1 s of delay ([0.5s,1.5s]), which was available on all trials: (1) mean power modulation (*pow*) in the range 5–35 Hz, (2) mean decoding precision (*dp*), (3) across-trial variance (*atv*) of the time-averaged power modulation estimates, and (4) within-trial variance (*wtv*) of power modulation estimates across 20 successive time steps within individual trials, followed by averaging variance estimates across trials. For simplicity and due to a lack of a priori hypotheses about specific key regions, we averaged these markers across all 180 ROIs (see below, Definition of ROIs and ROI groups) and *z*-scored across included subjects.

We then fit different multiple linear regression models to predict different individual cognitive measures (model parameter estimates as well as cognitive integrity scores) from these four cortex-wide neural markers of delay activity:

(14) $$y=\;\beta_{0}+\beta_{1}\times \mathrm{dp}+\beta_{2}\times \mathrm{pow}+\beta_{3}\times \mathrm{atv}+\beta_{4}\times \mathrm{wtv}+\beta_{5}\times \mathrm{trl}+\epsilon ,$$

where *y* were, in different model fits, the parameter estimates from the behavioral model or individual cognitive integrity scores and trl were the individual trial counts (*z*-scored) that were included as nuisance regressor to account for interindividual differences in the trial counts available for MEG analyses. This analysis was performed including all subjects in a single model, as well as for OHC and MCI groups separately. We further investigated the relationship of the measures of temporal generalization to task-related behavioral measures and cognitive integrity using correlation analysis.

#### Definition of ROIs and ROI groups

We based the anatomical definition of ROIs on a multimodal MRI-based parcellation of the cerebral cortex (180 ROIs; Glasser et al., 2016). For certain analyses focusing on specific regions along the visuomotor cortical pathway, we collapsed results across multiple ROIs belonging to certain ROI groups (defined in the Supplement of Glasser et al., 2016) as described in Table 3.

Here, only the primary motor region was defined independently—on the basis of the previous work of our laboratory—as the hand-specific area (de Gee et al., 2017). For calculating whole-cortex maps of certain statistics, we used the 22 ROI groups defined in the supplementary text of Glasser et al. (2016).

### Eye-tracking data analysis

#### Preprocessing

Eye positions and pupil size were recorded during the performance of the working memory task and were preprocessed using customized scripts and FieldTrip (Oostenveld et al., 2011) for MATLAB following the approach used in our laboratory's previous work (Murphy et al., 2021). Two subjects (OHC, *N* = 1; UNC, *N* = 1) and five individual blocks from other subjects had to be excluded from the analysis of gaze directions due to technical problems during the recording. Blinks and missing data were linearly interpolated to obtain a continuous time series of gaze directions during task performance from which *x*- and *y*-gaze directions during the trials (−0.1–1.6 s with respect to sample stimulus onset) were extracted. Individual trials were excluded if >60% of the extracted trial data had to be interpolated, if a prolonged artifact occurred (>0.3 s of consecutive interpolation) and/or if the standard deviation of the recorded *x*-gaze directions within a trial was unfeasibly small (indicating loss of corneal reflection during the recording; threshold SD = 10^−6^).

Subjects whose data consisted of fewer than 60 trials after the preprocessing steps described above were excluded from any further analyses of gaze directions (Fig. 1*A*). On average (SD), 136.76 (34.91) trials per subject were submitted to further analyses (range across subjects, 63–189 trials). For comparison with the younger subjects, the eye-tracking data of the older subjects were resampled to the same sampling rate as in Dataset 2 (250 Hz).

#### Decoding of sample spatial location from gaze direction

We focused the gaze direction analysis on the time from the stimulus onset to the end of a delay duration of 1 s. The Cartesian coordinates (*x* and *y*) of gaze directions provided by the eye tracker were used as features for training classifiers, which were identical to the decoding analysis from the MEG activity described above, with the exception that no dimensionality reduction needed to be performed (because there were only two features).

### Statistical analysis

Within-subject tests were nonparametric permutation tests against zero (10,000 permutations). For comparisons between groups of subjects, we used between-subject nonparametric permutation tests (10,000 permutations). All permutation tests were performed two-sided, the only exception being within-subject statistics of time-resolved (identical train and test time) decoding precision where we specifically tested for the presence of above-chance decoding.

Deviations from zero in ROI- or ROI-group-specific TFRs and decoding time courses were identified through cluster-based permutation testing (Maris and Oostenveld, 2007; 10,000 permutations, cluster-forming threshold, *p *< 0.05). Maps of measures plotted over the entire cortex (brain maps) were thresholded through FDR correction at a significance threshold of *p *< 0.05.

All correlation analyses were performed using Pearson’s correlation (two-tailed).

Evidence for the null hypothesis was tested by estimating the Bayes factor (BF_10_) from a *T* statistic (Krekelberg, 2022).

#### Code accessibility

Analysis code is available at https://github.com/DonnerLab/2024_Monov_WM-dynamics-in-the-aging-human-brain.

#### Data availability

For reasons of data protection, raw behavioral data and preprocessed MEG data can be made available upon request.

## Results

We used an integrative approach to gain insight into aging effects on the stability of working memory representations and to relate these to clinical measures of cognitive integrity (Fig. 1*A*). Through behavioral modeling, we quantified working memory dynamics in terms of latent variables. For all older participants, we measured their cortical population activity using MEG and their performance in a neuropsychological test battery. We combined categorical and dimensional approaches in our analysis of working memory mechanisms by (1) comparing model- and MEG-based measures between groups (OHC, MCI, and—as a reference for behavioral modeling—YHC) and (2) for the older participants, correlating our measures with a clinical assessment of their cognitive integrity. All participants in the MCI group were classified as amnestic in neuropsychological testing (Table 1).

The first principle component (PC1) of the scores from the CERAD-Plus test battery (Moms et al., 1989; Mirra et al., 1991; Schmid et al., 2014) consisted of positive contributions of performance scores in all tests (Fig. 1*B*, left, inset) and explained 42.5% of variance in the test data (Fig. 1*B*, left). Word list learning, the subtest in which performance can be most straightforwardly linked to the integrity of working memory, held the highest loading of all CERAD-Plus subtests on PC1 (∼0.41; Fig. 1*B*, inset); and there was a significant difference in performance on this subtest between OHC and MCI (Extended Data Fig. 1-1*A*, Extended Data Table 1-1), which may reflect the diagnostic importance of working memory in MCI. The eigenvalues (scores) associated with PC1 were (1) strongly correlated to alternative summary metrics [CERAD “total score” (Chandler et al., 2005), Fig. 1*B*, middle, and MMSE (Folstein et al., 1975; Mitchell, 2009), Extended Data Fig. 1-1*B*] and (2) highly predictive of the clinical MCI diagnosis [receiver operating characteristic (ROC) value, ≈0.86, Fig. 1*B*, right]. In the following, we used these scores (denoted as “PC1 score”) as a summary measure to quantify each older individual's cognitive integrity.

### Impaired working memory performance in older adults

In our delayed match-to-sample working memory task, subjects judged whether a “sample” and a “test” stimulus had the same or a different spatial location (Fig. 1*C*). As expected from previous work (Nilsson and Nelson, 1981; Zhang and Luck, 2009; Shin et al., 2017), the difficulty of this judgment depended on both temporal and spatial distances between the sample and the test (Fig. 2*A,B*; Extended Data Fig. 2-1*A,B*), with the error rate increasing with delay duration (collapsing across match and nonmatch trials; main effect of delay in mixed delay_ _*_ _group ANOVA: *F*_2,126 _= 38.6; *p *< 10^−4^; Fig. 2*A*) as well as with test-sample distance (nonmatch trials only; main effect of distance in mixed distance_ _*_ _delay_ _*_ _group ANOVA: *F*_1,63 _= 258.84; *p *< 10^−4^; Extended Data Fig. 2-1*B*).

10.1523/JNEUROSCI.1883-23.2024.f2-1 Figure 2-1 **Error rates and model predictions on working memory task differentiated by delay duration, sample-test stimulus distance, and participant group. (A)** Miss rates (mean ± s.e.m.) by delay duration for YHC (yellow, N = 21), OHC (blue, N = 20) and MCI (red, N = 19). **(B)** False alarm rates (mean ± s.e.m.) on near non-match trials (lighter shades) and far non-match trials (darker shades). **A**&**B** Model predictions (mean ± s.e.m.) are shown as shadings in the corresponding color. Download Figure 2-1, TIF file.

Group-wise comparisons of error rates (two-sided permutation tests) showed significant differences in the performance between YHC and MCI (*p *= 0.0061), while there were no significant group differences between YHC and OHC (*p *= 0.0707) or OHC and MCI groups (*p *= 0.45; Extended Data Fig. 1-1*C*). Previous modeling of spatial delayed match-to-sample performance showed that false alarms on trials with small test-sample distance are particularly informative about the stability of working memory representations (Murray et al., 2014). Indeed, while we observed no effects of group on misses and false alarms for “far” trials (Extended Data Fig. 2-1*A,B*), younger healthy controls performed better than older adults on the near false alarm trials, for both 1 and 3 s (but not 9 s) delays (Fig. 2*B*, left and middle; planned comparisons, two-sided permutation tests). There was a similar, but only trending, effect for an increased task accuracy of OHC compared with MCI on 9 s near trials (Fig. 2*B*, right).

### Model-based dissection of working memory performance

The behavioral effects reported above may, in principle, be due to changes in the stability of the working memory representation or in the response strategy of the subjects. Both factors may change with age. To disentangle these possibilities and to gain deeper mechanistic insight, we developed a model of working memory dynamics inspired by previous cortical circuit modeling work (Compte, 2000; Engel and Wang, 2011; Murray et al., 2014). These biophysically detailed models consist of many parameters, which preclude fitting them to behavioral data in a principled fashion. To estimate individual parameters quantifying the mechanisms governing working memory performance, we therefore opted for a more abstract (“algorithmic”) modeling approach using a small set of free parameters that were sufficiently constrained by our behavioral data.

Our approach assumed that instability of a working memory representation may originate from two sources. First, a random drift of the activity pattern in the neuronal population encoding the sample stimulus (i.e., spatial location) will introduce a random error in the information encoded at the end relative to the start of the delay period (Murray et al., 2014; Wimmer et al., 2014; Schneegans and Bays, 2018). This was captured by describing the sample representation during delay as a particle subject to a random diffusion process (Fig. 3*A*, left; Panichello et al., 2019; Schapiro et al., 2022) with a diffusion constant that captured the memory noise. Second, the activity pattern may vanish altogether before the end of the delay period (Zhang and Luck, 2009). In the simplest model variant tested, this was captured by a single lapse parameter (see below and Materials and Methods). For the same/different judgment required by our task, the model computed the absolute distance between memory representation at the end of delay and the then-shown test stimulus, resulting in the decision variable. The judgment was then produced through the application of a threshold (hard cutoff or smooth function; see below) to this decision variable (Fig. 3*A*, middle).

10.1523/JNEUROSCI.1883-23.2024.f3-1 Figure 3-1 **Model validation and comparison. (A)** Histograms of fitted parameters for simulated data sets (models 1-6, colored), the corresponding fit of a normal density function (solid black line) and parameter levels at which the data sets were simulated (dashed black line). **(B)** Width of the probability density function for fitted model parameters that are included in all candidate models (memory noise and decision threshold) at half maximum. Models 4 and 6 (including decision noise as a free parameter) showed the strongest limitations in the recoverability of memory noise and threshold. **(C)** Comparison of the candidate models 1-6 from the mean BIC scores (*right y-axis*) and the BIC score relative to the winning model (*left y-axis*) for MCI patients (red) and the entire sample (black), indicating superiority of Model 2. **(D)** Intercorrelation and histograms of fitted model parameters for Model 2 across all subjects (N = 67). **(E)** Model predictions of Model 2 were tested for match (*top*) and non-match trials (*bottom*) separately. Circles represent predicted probability of a “different” response on 1, 3 and 9  s delay trials per subject (N = 67) as a function of the corresponding observed proportion of behavioral responses for the same trial-type. Non-match trials were segregated into “far” and “near” trials analogous to behavioral analyses. Statistics refer to linear regression model fits of predicted and actual responses. Grey solid lines represent the identity line. **(F)** Correlations of fitted model parameters (N = 67) with standard SDT measures of criterion (c) and sensitivity (d’) further serve as validation of fitted model parameters. Download Figure 3-1, TIF file.

10.1523/JNEUROSCI.1883-23.2024.f3-2 Figure 3-2 **Relationship of memory noise and threshold parameters. (A)** Memory noise levels and corresponding optimal threshold fits are strongly positively correlated in simulated data showing an optimality interdependence of the two parameters. **(B)** Correlation of working memory accuracy with fitted noise (*top*) and threshold (*bottom*) parameters for all subjects (black, N = 67), YHC (yellow, N = 21) OHC (blu*e*, N = 20) and MCI (red, N = 19). Across all participant groups, high noise parameters are related to decreased accuracy on the working memory task. Such a relationship with threshold parameters is present across the entire sample, however on a subgroup level it can only be found in the older subjects. There is no significant difference in the correlation coefficients between YHC and older subjects or either of the older participant groups separately (two-sided permutation tests on Δr, all *p *> 0.14). Download Figure 3-2, TIF file.

We fit the behavioral data with a selection of model variants differing in the composition of free parameters (see Materials and Methods). Model validation with synthetic data as well as model comparison (Table 2; Extended Data Fig. 3-1*A–C*) favored a model variant containing the following free parameters: memory noise (*σ*_mem_), decision threshold (*δ*), and lapse probability (*θ*). Here, the decision translating the difference between the memory representation and test stimulus locations into a same/different response was modeled as a deterministic process (i.e., step function). The probability of lapses (i.e., random responses) was reflected in symmetric asymptotes of this function (Fig. 3*A*, middle). The model yielded probabilities of “different” judgments as a function of delay and sample-test stimulus distance (Fig. 3*A*, right). We used this model variant for all analyses described below.

The predictions of the fitted model were largely consistent with participants’ performance for all participant groups and conditions assessed here (Fig. 2*A,B*; Extended Data Fig. 2-1*A,B*). Analysis of the overall goodness of fit of the behavioral model showed good correspondence between model predictions and subjects’ responses for both match and nonmatch trials [match trials: *R*^2^(adjusted) = 0.7022, *p*(*F*-test) < 10^−4^, nonmatch trials: *R*^2^(adjusted) = 0.8425, *p*(*F*-test) < 10^−4^; Extended Data Fig. 3-1*E*]. Further, we found expected correlations of the fitted model parameters with model-free metrics of behavioral performance based on signal detection theory: threshold *δ* was positively correlated with signal detection theoretic criterion *c* (Extended Data Fig. 3-1*F*, *left*), and both memory noise (*σ*_mem_) and lapse (*θ*) parameters were negatively correlated with sensitivity *d*′ (Extended Data Fig. 3-1*F*, middle and right; all Pearson’s correlations, *p *< 10^−4^).

The lapse parameter in this model variant captured lapses occurring at different levels of processing (sensory encoding, memory maintenance, decision, and action selection), which were not dissociable in the current task. We further note that, while the DF is certainly an oversimplification, model comparison did not favor a model with a noisy decision transformation (Extended Data Fig. 3-1*A–C*), likely because the data also did not allow for disentangling behavioral variability due to lapses versus decision noise.

The model revealed smaller memory noise (*σ*_mem_) for young healthy adults compared with MCI patients and a trending effect compared with the OHCs (Fig. 3*B*, left; one-way ANOVA noise: *F*_2,57 _= 2.76; *p* = 0.07). We combined memory noise and lapse probability into a single measure that captured all stochasticity in internal processing distinct from strategic sources of error (i.e., decision threshold *δ*). This revealed larger behavioral stochasticity in both groups of older adults than the younger healthy controls (Fig. 3*B*, third from left; one-way ANOVA noise + lapse: *F*_2,57 _= 3.25; *p* = 0.0461). There was no evidence for group differences in other model parameters (decision threshold, lapse probability on its own; Fig. 3*B*; one-way ANOVA lapse: *F*_2,57 _= 1.03, *p* = 0.36; one-way ANOVA threshold: *F*_2,57 _= 0.58, *p* = 0.56). In order to assess whether age-related working memory deteriorations are specifically due to the decreased quality of working memory representations rather than changes in task strategy (i.e., fitted threshold parameters), we quantified the strength of evidence in support of the hypothesis that threshold parameters do not differ between groups. This revealed only weak evidence for the null hypothesis [BF_10_(YHC/all older) = 0.42, *p *= 0.31; BF_10_(YHC/MCI) = 0.46, *p *= 0.32; BF_10_(YHC/OHC) = 0.39, *p *= 0.45]. We also assessed the statistical significance of the difference between the group effects on the behavioral stochasticity and threshold parameters (Δdiff) using a two-sided permutation test on the normalized data. We did not find support for the specificity of group differences in the fitted stochasticity parameters compared with fitted threshold parameters [Δdiff(YHC/all older) = −0.4255, *p *= 0.1182; Δdiff(YHC/MCI) = −0.4638, *p *= 0.1852; Δdiff(YHC/OHC) = −0.3936, *p *= 0.2284; Δdiff(OHC/MCI) = 0.0497, *p *= 0.8960]. Thus, although we found statistically significant effects of group on the stochasticity but not threshold parameters, we did not find conclusive evidence that the age-related deterioration in task performance can be ascribed only to increased stochasticity.

Furthermore, we observed that the parameter estimates for *σ*_mem_ were correlated, across subjects, to those for *δ* (Pearson’s correlation, *r *= 0.25, *p *= 0.04; Extended Data Fig. 3-1*D*). We reasoned that this correlation may be genuine, reflecting a strategic adaptation to participants’ individual levels of memory noise, applying higher thresholds for “different” decisions (i.e., higher values for *δ*) for higher memory noise (increased *σ*_mem_). To evaluate the plausibility of this idea, we assessed whether such a strategic threshold adjustment would help to maximize performance under larger levels of *σ*_mem_. Through simulation (three task blocks = 189 trials), we computed the *δ* settings that lead to the highest accuracy for 100 different levels of *σ*_mem_ covering the range of fitted noise parameters observed in the subjects of the study. The performance-maximizing *δ* values were identified using simplex search (D’Errico, 2012) minimizing the objective: 1−P(correct), where P(correct) is the average response accuracy across trials. Indeed, a more conservative threshold (higher *δ*) produced better task performance when memory noise was high (Pearson’s correlation, *p *< 10^−4^; Extended Data Fig. 3-2*A*). To further investigate the notion of strategic threshold adjustments, we performed another control analysis in which we correlated working memory accuracy with memory noise as well as threshold parameter fits (Extended Data Fig. 3-2*B*). As expected, this showed that memory noise has a consistent negative effect on working memory accuracy across all subgroups (all subjects, YHC, OHC, MCI). Interestingly, for fitted threshold parameters, negative correlations with working memory accuracy were instead only present in both older groups (OHC, MCI), but not in the younger subjects (Extended Data Fig. 3-2*B*). Our data therefore point to possible strategic threshold adjustments in aged individuals as an optimal strategy to remediate task performance in the face of increased noise levels, which can readily be related to the literature (Ratcliff and McKoon, 2008).

In sum, the group differences in behavioral performance of the working memory task were related to the age-related deterioration of the stability of the working memory representation, which in turn might lead to (potentially compensatory) strategic changes in decision thresholds.

### Relating working memory mechanisms to cognitive integrity

We next used a dimensional approach focusing on individual differences to link the working memory mechanisms to cognitive integrity. Cognitive aging is characterized by substantial differences between individuals, which are only partly captured by the clinical classification into OHCs and MCI categories (Fig. 1*A,B*). For example, seven older participants who were originally recruited for our OHC group exhibited neuropsychological test scores comparable with MCI and were, therefore, classified as a separate cohort for the purpose of this study (UNC; Fig. 1*A*). In the following, we used each participant's neuropsychological test results, summarized by the eigenvalue of the PC1 (Fig. 1*B*), as an individual measure of overall cognitive integrity.

**Figure 4.**
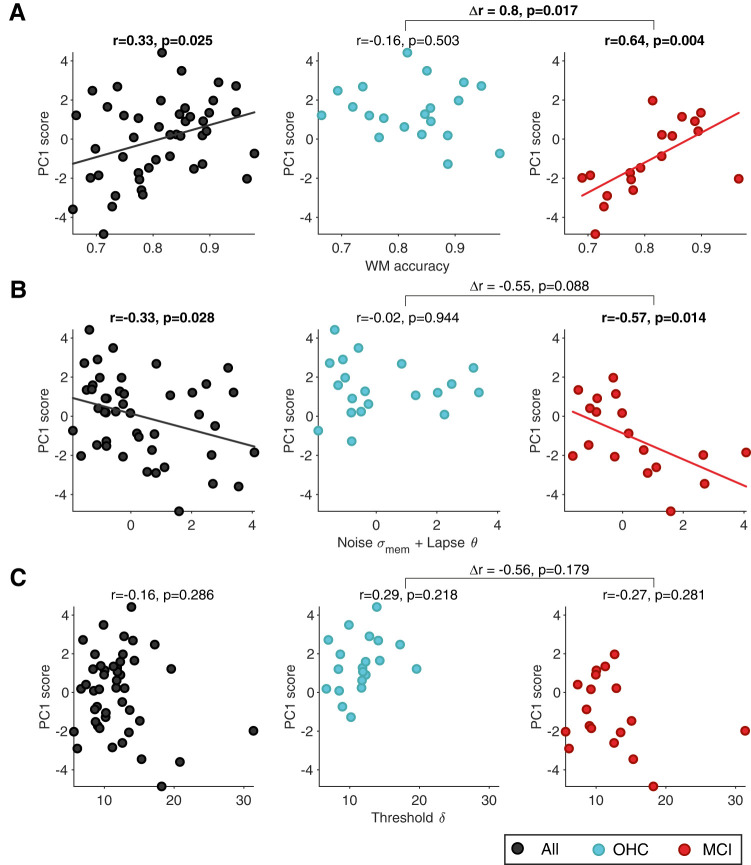
Correlation of cognitive integrity and working memory mechanisms. Correlation of PC1 scores (for quantifying overall cognitive integrity, see Fig. 1*B*) with (***A***) working memory (WM) task accuracy, (***B***) combination of memory noise and lapse probability fits, and (***C***) threshold fits. In all panels, circles represent individual subjects within the group of older participants. Correlations and correlational statistics are reported for all older subjects (*N* = 45, black, left), OHC (*N *= 20, blue, middle) and MCI (*N* = 18, red, right) separately. The size of the complete sample of older participants (“All”) was larger than the sum of OHC and MCI sample sizes because a subset of older participants could not be classified in either category (see Materials and Methods). Linear regression fit is shown for statistically significant correlations only. Differences in correlations between OHC and MCI are shown on top of the square brackets; associated *p*-values refer to two-sided permutation tests. See Extended Data Figure 4-1*A* for correlation of PC1 scores with memory noise and lapse probability alone and Extended Data Figure 4-1*B* for the same correlational analyses performed using CERAD total scores instead of PC1 scores.

Individual cognitive integrity was robustly correlated to performance on the working memory task as well as the model parameters capturing behavioral stochasticity (in particular, stability of memory representation): higher cognitive integrity was associated with higher task accuracy and lower combined memory noise/lapse scores from the model (Fig. 4*A,B*). Remarkably, the correlation with task accuracy was present only in the MCI but not the OHC group individually, with a clear difference in correlation between the two groups (Fig. 4*A*). Likewise, there was a correlation to cognitive integrity for noise and lapse probability combined in the MCI but not the OHC group, with a trend toward a difference in correlations between the groups (Fig. 4*B*). This correlation in the MCI group was primarily driven by memory noise over lapse probability (Extended Data Fig. 4-1*A*). By contrast, we observed no correlation between cognitive integrity and the decision threshold parameter (Fig. 4*C*). The same correlational analyses performed using the CERAD total score (Chandler et al., 2005) yielded comparable results (Extended Data Fig. 4-1*C*).

In sum, we established a link between individual cognitive integrity on the one hand and working memory task performance as well as model-estimated memory noise parameters on the other hand, specifically in the MCI group. Our final analyses aimed to relate the modeling results to direct measurements of cortical delay activity underlying the working memory task.

10.1523/JNEUROSCI.1883-23.2024.f4-1 Figure 4-1 **Correlation analyses with cognitive integrity scores.** Correlation of PC1 scores with **(A)** noise parameter fits and **(B)** lapse parameter fits. In all panels, circles represent individual subjects within the group of older participants. Correlations and correlational statistics are reported for all older subjects (N = 45, black, *left*), OHC (N = 20, blue, *middle*) and MCI (N = 18, red, *right*) separately. Linear regression fit is shown for statistically significant correlations only. Differences in correlations between OHC and MCI are shown on top of the square brackets, *p*-values refer to two-sided permutation tests. **(C)** Correlational analysis for behavioral measures derived from the WM task with established CERAD total scores (analogous to analysis with PC1 scores). Bar graphs depict Pearson’s correlation coefficient with the corresponding *p*-value on top of each bar. Results are expectedly similar to the same correlations with PC1 scores (Fig. 4). Download Figure 4-1, TIF file.

### Task-related cortical dynamics and link to behavior

**Figure 5.**
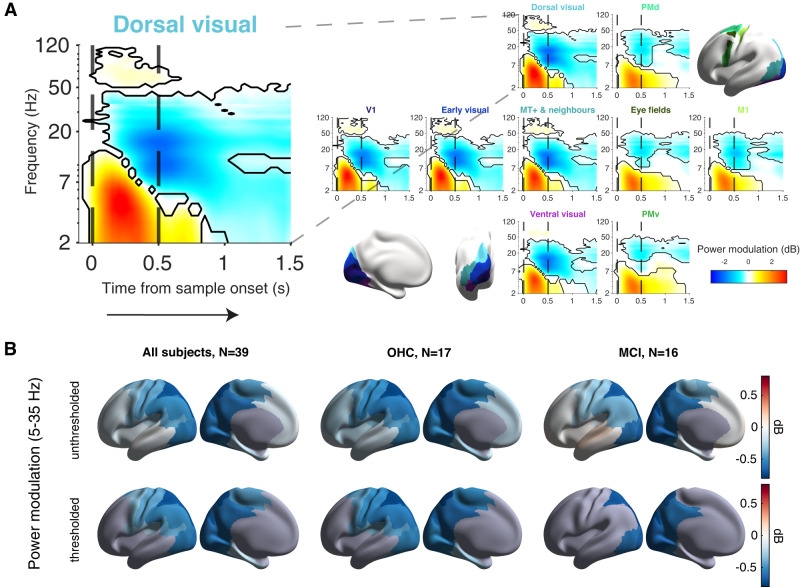
Cortical population dynamics during working memory task. ***A***, TFRs of task-induced power modulations across cortical visuomotor pathway. Data are shown from 0.1 s before sample onset (first vertical black dashed line) until 1 s after sample offset (second black dashed line) and collapsed across all older adults (*N* = 39). Black contouring captures time-frequency clusters of significant within-subject power modulations (*p *< 0.05, cluster-based permutation test). Brain maps at inset depict clustered brain regions of interest (Table 3), colored to match panel titles of heat maps (***A***). ***B***, Maps of low-frequency power modulation (averaged over 5–35 Hz and [0.5, 1.5 s]). Left to right: all older participants (*N* = 39), OHC (*N *= 17), and MCI (*N* = 16). Top, not thresholded; bottom, statistically thresholded difference maps (*p *< 0.05, FDR-corrected). See Extended Data Figure 5-1*A* for TFRs of power modulations for longer delay trials and Extended Data Figure 5-1*B* for equivalent fine-grained brain maps.

The combination of spectral and source analysis with anatomical atlases (Wilming et al., 2020; Murphy et al., 2021) yielded a detailed description of the task-related cortical population dynamics (Fig. 5). The test stimulus induced a transient increase in the <8 Hz and 50–100 Hz (gamma) frequency ranges in visual cortical areas, followed by suppression in the 8–36 Hz (alpha/beta) frequency range (Fig. 5*A*; Extended Data Fig. 5-1*A*). This matches the general pattern of visual stimulus-induced MEG power modulations (Donner and Siegel, 2011), including studies using similar visual stimuli (Murphy et al., 2021), so it served as a quality control of our MEG data.

10.1523/JNEUROSCI.1883-23.2024.f5-1 Figure 5-1 **Power modulation on longer delay trials and brain maps for fine-grained cortical parcellation.** **(A)** Task-induced power modulations for dorsal visual clustered brain region (see Table 3) for 3  s delay (including first three seconds of trials with a 9  s delay duration) and 9  s delay show the persistence of power suppression with relative omission of alpha frequencies beyond the first second of delay (all subjects, N = 39). Black contouring refers to significant within-subjects power modulation (cluster-based permutation test, *p *< 0.05). **(B)** Fine-grained parcellated brain maps (N = 180 ROIs) of unthresholded (*top*) and FDR-corrected (*bottom*) low-frequency (5-35  Hz) power modulation during first second into delay duration (significance threshold, *p *< 0.05). Download Figure 5-1, TIF file.

Importantly, the low-frequency power suppression was sustained throughout the delay interval (Fig. 5*A*; Extended Data Fig. 5-1*A*) and widely distributed across the posterior cortex (Fig. 5*B*; Extended Data Fig. 5-1*B*). The magnitude and spatial distribution of this power suppression during delay were similar in the OHC and MCI groups, without any evidence for differences (Fig. 5*B*; Extended Data Fig. 5-1*B*).

**Figure 6.**
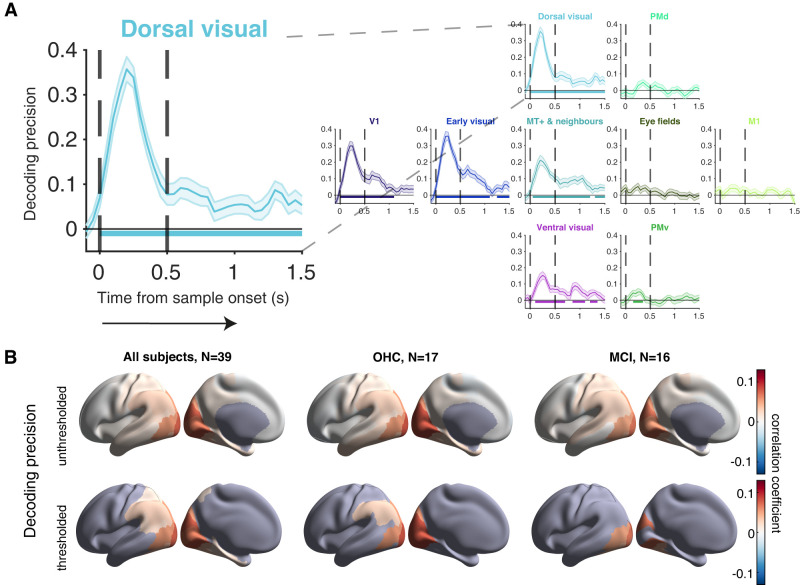
Cortical dynamics during working memory task. ***A***, Time courses of vertex-based sample location decoding precision (correlation coefficient between the predicted sample angle and its actual angle) for clustered brain regions across the visuomotor pathway (all older subjects, *N* = 39; mean ± SEM). Times of sample onset and offset are marked by vertical black dashed lines. Colored horizontal lines indicate latencies of significant decoding precision (*p *< 0.05, cluster-based permutation test). ***B***, As Figure 5*B*, but for decoding precision during delay ([0.5–1.5 s]). See Extended Data Figure 6-1*A* for decoding precision on 3 s delay trials and encoding of sample hemifield. Equivalent fine-grained brain maps are presented in Extended Data Figure 6-1*B*.

We used spatiospectral features (Materials and Methods and Wilming et al., 2020) to decode the spatial location of the sample in the local cortical population activity (Fig. 6). While the sensory response produced the highest decoding precision during sample presentation, all visual cortical areas continued to show above-chance decoding during delay, most pronounced and sustained in the dorsal visual cortex (Fig. 6*A,B*; Extended Data Fig. 6-1*A,B*). Like the power modulations, decoding precisions were similar for both groups (Fig. 6*B*; Extended Data Fig. 6-1*B*). We were unable to detect sustained stimulus encoding beyond the shortest delay interval of 1 s—possibly due to highly limited trial counts for these longer trials—and a more coarse-grained approach, decoding the hemifield in which the sample stimulus was presented, yielded largely equivalent results (Extended Data Fig. 6-1*A*).

10.1523/JNEUROSCI.1883-23.2024.f6-1 Figure 6-1 **Decoding precision of hemifield and exact sample location on longer delay trials and brain maps for fine-grained cortical parcellation.** **(A)** Encoding of sample hemifield (*orange*) vs. exact sample location (*blue*) in dorsal visual cortex. Time courses of vertex-based decoding precision (power values from 5-35  Hz; all older subjects, N = 39; mean ± s.e.m.) for encoding of the exact sample location (correlation coefficient of predicted and actual location; *left y-axis*) and the sample hemifield (area under the ROC curve from prediction scores for classification of hemifield; *right y-axis*) for 1  s delay duration (*left*) and 3  s delay duration (*right*). Dashed lines represent sample stimulus onset and offset. Solid horizontal lines in the corresponding color represent latencies of significant decoding precision (cluster-based one-sided permutation test against zero for correlation coefficients or against 0.5 for AUC, *p *< 0.05). Although dorsal visual cortex showed robust encoding when considering only the first second of the delay period of all trials, no significant decoding precision could be demonstrated throughout the 3  s delay duration. Decoding of the exact location and the sample hemifield yield comparable results. **(B)** Fine-grained parcellated brain maps (N = 180 parcels) of unthresholded (*top*) and FDR-corrected (*bottom*) decoding precision during first second into delay duration (significance threshold, *p *< 0.05). Download Figure 6-1, TIF file.

Another set of our analyses related the behavioral modeling parameters to different markers of cortical delay activity during our task. We quantified four measures of the delay activity (from 0.5 to 1.5 s): in addition to pow (5–35 Hz) and dp (as shown in Figs. 5, 6) and also the within- and across-trial variability of these power modulations (Extended Data Fig. 7-1*A,B*). These measures were included because neural variability has been implicated in cognitive integrity and aging (Grady, 2012; Dinstein et al., 2015; Garrett et al., 2021; Waschke et al., 2021). Due to the widespread distribution of decoding precision and overall power modulation, we collapsed each measure of delay activity across all cortical ROIs (*N* = 180). We then fit a series of linear models regressing individual cognitive performance measures onto these activity markers (alongside some nuisance regressors).

**Figure 7.**
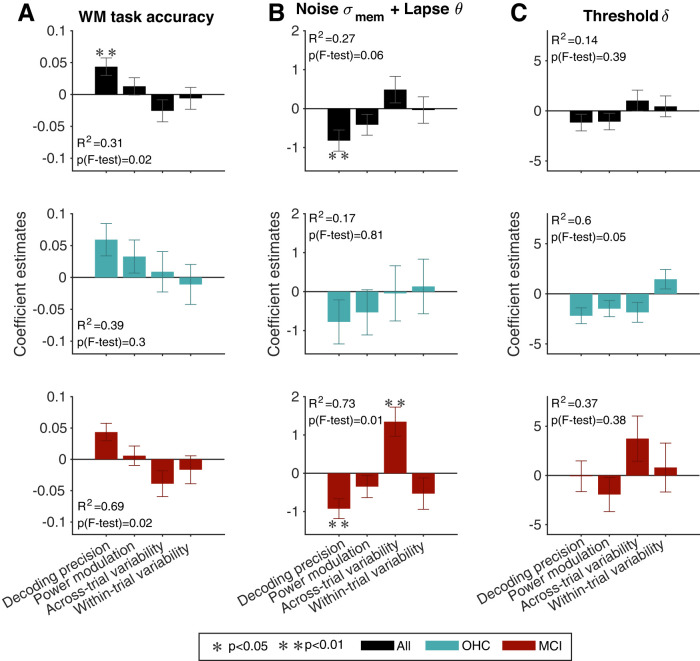
Linking markers of cortical delay activity to working memory stability. Coefficient estimates (±standard error) from multiple linear regression models predicting (***A***) working memory accuracy, (***B***) the combination of memory noise and lapse parameters, and (***C***) threshold across older individuals (*N* = 39; black), OHC (*N* = 17; blue), and MCI (*N* = 16; red) using standardized versions of the four neural measures (*x*-axis) evaluated across the entire cortex. *, *p *< 0.05; **, *p *< 0.01 (FDR-corrected, nonsignificant otherwise). See Extended Data Figure 7-1 for the cortical distributions of the neural markers across-trial variability and within-trial variability.

These neural markers were not linked to individual cognitive integrity (PC1 score; all *p *> 0.05). But higher neural decoding precision predicted higher working memory task accuracy across subjects (similar trends within each group; Fig. 7*A*). None of the three other neural measures showed such a relationship. This effect was accounted for by a link between decoding precision and behavioral stochasticity parameters (memory noise and lapse combined; Fig. 7*B*), but not decision threshold (Fig. 7*C*), again with a similar trend within each group (significant for MCI, but not OHC).

10.1523/JNEUROSCI.1883-23.2024.f7-1 Figure 7-1 **Cortical distributions of variability of task-related MEG power.** **(A)** Maps of across-trial variability in power modulations during 1  s of delay duration in the frequency range of 5-35  Hz. **(B)** As A, but for within-trial variability in power modulations. Download Figure 7-1, TIF file.

Notably, the analysis also revealed across-trial variability of power modulations as a second significant predictor of individual differences in the behavioral stochasticity parameters (memory noise and lapse probability combined), specifically in the MCI group (Fig. 7*B*), and again with no effect for threshold (Fig. 7*C*).

**Figure 8.**
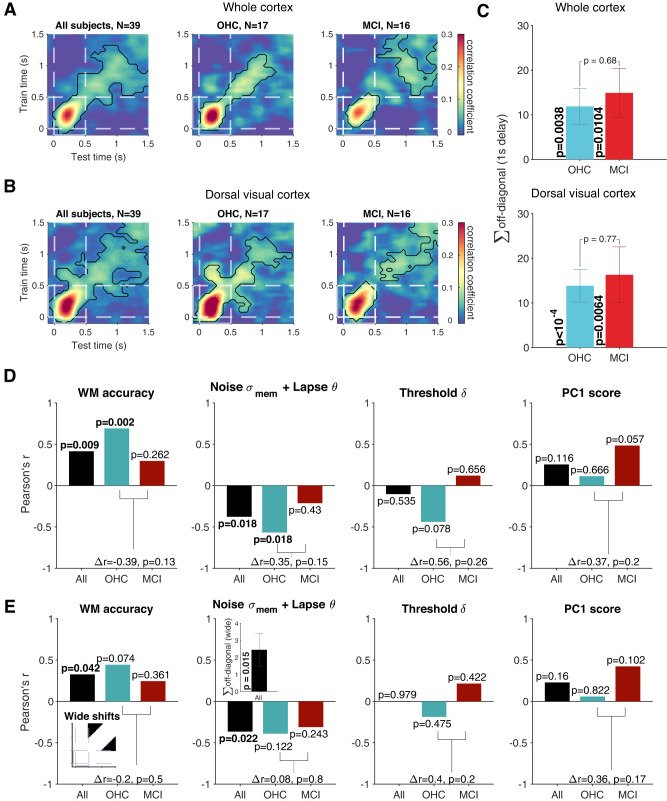
Temporal generalization of working memory code. ***A***, Cross-temporal decoding precision (correlation coefficient between predicted and actual sample stimulus angles) using the vertices across the whole cortex up to 1 s of delay duration for (left to right) all subjects, OHC, and MCI patients. White dashed lines depict memorandum onset and offset. Black contouring captures clusters of significant decoding precision (within-subject cluster-based permutation test, *p *< 0.05). ***B***, Same as ***A***, but for the dorsal visual cortex only. ***C***, Sum of all off-diagonal elements (mean ± SEM) during the working memory delay (1 s), for OHC (blue) and MCI (red) across the whole cortex (top) and in the dorsal visual cortex (bottom). *P*-values correspond to within-subject permutation tests against zero (side of each bar graph) and between-subject permutation tests (top of square brackets). ***D***, Correlation coefficients of off-diagonal-sum of cross-temporal decoding precision in the dorsal visual cortex with behavioral measures. Left to right: working memory accuracy, fitted behavioral stochasticity parameters (memory noise and lapse probability), fitted threshold parameter, and PC1 scores. ***E***, Same as ***D***, but for sum of wide-shifted off-diagonal elements. Insets: Left: depiction of included elements (black) for off-diagonal sum with wide shifts between train and test time. Left–middle: wide-shifted off-diagonal sum in the dorsal visual cortex for all older participants (*N* = 39; mean ± SEM). *P*-value at the side of the bar graph corresponds to a permutation test against zero (two-sided). See Extended Data Figure 8-1 for tests of the dynamic working memory code.

Finally, we analyzed the temporal generalization of decoding of the sample stimulus location to test the extent to which the sustained above-chance decoding identified in the previous analysis (Fig. 6*A*) reflected a stable mnemonic code. Examining the temporal generalization matrix, we found statistically significant off-diagonal decoding precisions during the delay period across the whole cortex (Fig. 8*A*) as well as specifically in the dorsal visual cortex (Fig. 8*B*), suggesting some stability of the memory code over time. However, in line with some previous findings (Trübutschek et al., 2017), this temporal generalization was also not perfect—that is, we did not observe a uniform square of above-chance decoding precisions in the temporal generalization matrix encompassing the delay, which would be indicative of a code that was perfectly stable over time.

10.1523/JNEUROSCI.1883-23.2024.f8-1 Figure 8-1 **Testing dynamic working memory code.** Averaged temporal generalization matrices of all older subjects (N = 39) in dorsal visual cortex. Dashed white lines depict sample stimulus onset and offset. White contouring captures time points of dynamic coding (significance threshold, *p *< 0.05, **(A)**: unthresholded; **(B)**: after FDR-correction). Download Figure 8-1, TIF file.

To obtain a scalar metric of temporal generalization, we computed the sum across all off-diagonal elements of the temporal generalization matrix covering the delay period (1 s). Analysis of this metric showed that both groups exhibited significant above-chance off-diagonal decoding performance across the whole cortex as well as the dorsal visual cortex specifically, while the group comparison of this metric did not yield group differences between OHC and MCI (Fig. 8*C*).

We focused our further analyses on the dorsal visual cortex as this area showed significant decoding precision throughout the 1 s delay period (Fig. 6*A*). We did observe that the summed decoding precision of the off-diagonal elements in the dorsal visual cortex correlated positively across participants with working memory accuracy and negatively with fitted stochasticity parameters (Fig. 8*D*). At the subgroup level, this effect was only present for OHC, with no significant difference in these correlations between OHC and MCI (Fig. 8*D*). To increase the specificity of the scalar measure as an index of temporal generalization of the neural code and rule out confounding smoothing effects of our window for spectral analysis, in a separate analysis, we only included those matrix elements in the summation for which train and test time were further apart (Fig. 8*E*, left, inset). Across all subjects, we found significant encoding of the sample stimulus location during the delay (Fig. 8*E*, second from left, inset) and the same correlative relationship to task-related behavioral measures (Fig. 8*E*). In a final set of complementary analyses, we did not find evidence for a dynamic working memory code during the delay period, but did find evidence for a dynamic code during sensory encoding of the sample stimulus (Extended Data Fig. 8-1*A,B*). Thus, our data suggest that stability of the neural code may at least partially underlie maintenance of spatial location in working memory on our task.

Taken together, our MEG results support the notion that the behavioral model-derived parameters are useful markers of the neural mechanism of working memory and point to the potential relevance of trial-to-trial variability of power modulations as a marker of MCI.

### Decoding working memory content from gaze direction

Participants were instructed to keep their gaze fixated on the cross in the center of the screen during task performance. Even so, fixational eye movements are difficult to suppress (especially for individuals not used to psychophysical testing), and previous work has lined fixational eye movements to working memory performance (Van Ede et al., 2019; Willeke et al., 2019; Linde-Domingo and Spitzer, 2023; De Vries and Van Ede, 2024). Furthermore, even small fixational eye movements may affect E/MEG data (Yuval-Greenberg et al., 2008; Liu et al., 2022). For these reasons, we wondered if fixational eye movements may have had any relationship to behavioral task performance and/or influenced our MEG results.

**Figure 9.**
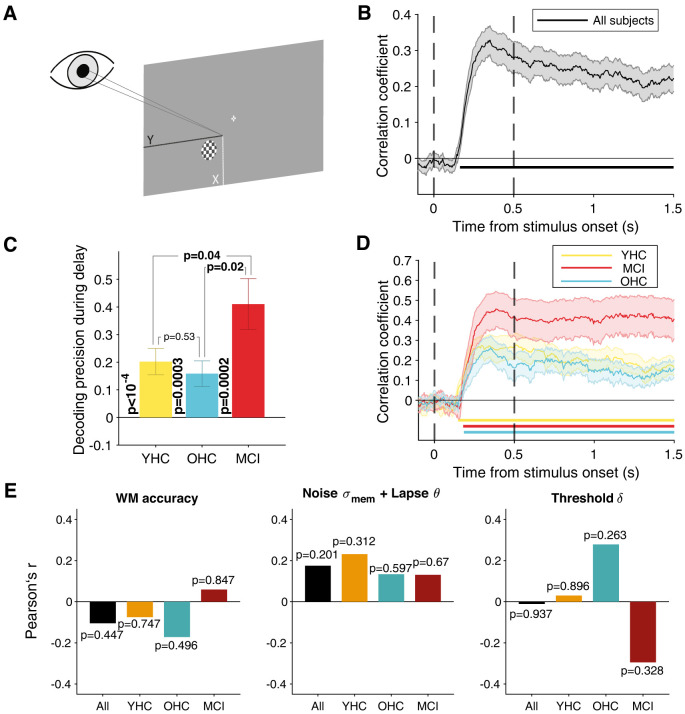
Decoding stimulus locations from gaze direction. ***A***, Illustration of the experimental setup with video eye tracker and depiction of measures characterizing the gaze direction relative to the fixation cross, *x*-gaze direction (white), and *y*-gaze direction (black). ***B***, Encoding of sample stimulus location (correlation coefficient of predicted and actual stimulus location) for 1 s delay duration across all subjects (*N* = 55). Solid horizontal line represents significant decoding precision (cluster-based one-sided permutation test against zero, *p *< 0.05). ***C***, Dp during 1 s delay for YHC (*N* = 21, yellow), OHC (*N* = 18, blue), and MCI (*N* = 13, red) groups. *P*-values on the side of each bar graph correspond to one-sided nonparametric permutation test against zero. *P*-values on top of the square brackets correspond to group-wise between-subject comparison of dp during 1 s delay. ***D***, Decoding precision time courses plotted for the subgroups YHC (*N *= 21, yellow), OHC (*N* = 18, blue), and MCI (*N* = 13, red) separately. Solid horizontal lines in the corresponding color represent significant decoding precision (cluster-based one-sided permutation test against zero, *p *< 0.05). ***E***, Between-participant correlation coefficients of dp with behavioral measures derived from the working memory task, with corresponding *p*-values on top of each bar graph for all subjects (black), YHC (*N *= 21, yellow), OHC (*N* = 18, blue), and MCI (*N* = 13, red). See Extended Data Figure 9-1 for decoding analysis for data sampled at 1,000 Hz (older subjects only).

We thus trained time-variant classifiers to predict the sample location from participants’ fixational eye movements (Fig. 9*A*, Materials and Methods). The sample stimulus location could be reliably decoded from gaze directions across all subjects during both sample stimulus presentation and the (1 s) working memory delay (Fig. 9*B*). This was also the case for each participant group (Fig. 9*C,D*; Extended Data Fig. 9-1*A,B*). There was also a significant difference in delay-period decoding precision between the MCI group and both younger and OHC groups (Fig. 9*C*; Extended Data Fig. 9-1*B*).

10.1523/JNEUROSCI.1883-23.2024.f9-1 Figure 9-1 **Analysis of gaze directions at higher temporal resolution (1000  Hz) for older subjects.** **(A)** Time courses of decoding precision from gaze direction data for MCI (red) and OHC (blue) groups separately. Dashed lines depict sample stimulus onset and offset. Solid horizontal lines in the corresponding color represent latencies of significant decoding precision (cluster-based one-sided permutation test against zero, *p *< 0.05). **(B)** Mean decoding precision during the delay period (1  s) for OHC (N = 18, blue) and MCI (N = 13, red). *P*-values on the side of each bar graph correspond to within-subjects non-parametric permutation tests against zero (one-sided). **(C)** Correlation coefficients of mean decoding precision with behavioral measures derived from the working memory task and PC1 scores with corresponding *p*-values on top of each bar graph for all subjects (N = 34, black), OHC (N = 18, blue) and MCI (N = 13, red). Download Figure 9-1, TIF file.

Importantly, we did not observe relationships between delay-period sample location decoding from gaze directions and behavioral/model-based measures from the working memory task (Fig. 9*E*; Extended Data Fig. 9-1*C*), nor the individual cognitive integrity scores of the older subjects (Extended Data Fig. 9-1*C*). Furthermore, the individual delay-period decoding precisions derived from the gaze data and those derived from the MEG data were not significantly correlated with each other in any of the defined ROI groups [*N* = 28, *p*-values ranging from 0.0537 to 0.9375 (mean, 0.5869); Fig. 6*B*].

In sum, while we found clear evidence for the sample stimulus location being encoded in gaze direction during the working memory delay, and differently so between groups, eye movements in our task were not directly related to behavioral accuracy or the precision of memory-related neural representations.

## Discussion

Age-related changes in working memory performance have been investigated using a variety of task protocols. This previous work has revealed diminished working memory capacity with age in contexts requiring maintenance of multiple memoranda (Bopp and Verhaeghen, 2018), especially when conjunctions of different object features (rather than single features) needed to be remembered (Peich et al., 2013; but see Pertzov et al., 2015). Less is known about the quality of working memory representations in human aging. One landmark study of aging monkeys has identified a deterioration of stimulus-selective persistent activity in the prefrontal cortex (M. Wang et al., 2011). It has remained unknown whether and how these neurophysiological changes generalize to the human brain and how they relate to working memory performance and to individual cognitive integrity. Our current study provides a comprehensive assessment of age-related changes in the stability of performance-relevant working memory representations and their neurophysiological bases, as well as their links to individual cognitive integrity in a clinically well-characterized sample of older participants.

One of the main insights afforded by our model-based approach is that reductions in working memory performance with aging (compared with younger adults) are related to a deterioration of the model-inferred stability (quality) of working memory representations and that this in turn is associated with individual differences in cognitive integrity within the group of older adults, establishing its broad functional relevance. These conclusions invite comparison with studies of age-related changes in perceptual decision-making (Ratcliff and McKoon, 2008). Some behavioral modeling work has revealed that the increases in reaction times in two-choice tasks commonly observed in older adults are not due to the less efficient integration of decision evidence, but to increased response caution, a strategic effect (Ratcliff and McKoon, 2008). Given the close mechanistic links between working memory and evidence accumulation in deliberative decisions (X-J. Wang, 2008; Schapiro et al., 2022), one might expect that the increase in memory noise we inferred in older adults will translate into less efficient evidence accumulation (i.e., lower drift rate; Ratcliff and McKoon, 2008), at least across longer timescales at which mechanisms of persistent activity play out. Indeed, recent work constraining behavioral model fits with neurophysiological data provided first evidence for degraded evidence accumulation in aging (McGovern et al., 2018). It will be instructive to study aging effects in evidence accumulation tasks that are more protracted (Tsetsos et al., 2012; Drugowitsch et al., 2016; Murphy et al., 2021) and/or more cognitive in nature (Purcell and Kiani, 2016; Van Den Brink et al., 2023) than the ones used in the existing aging literature.

The relationship between our behavioral markers of working memory quality (memory noise and lapse probability) and overall cognitive integrity was particularly pronounced in older individuals diagnosed with MCI and not in age-matched healthy controls. This indicates a tight link between deterioration of working memory mechanisms on the one hand and pathophysiological aging as gauged by standard comprehensive neuropsychological test batteries on the other hand. Likewise, the relationship between MEG markers of cortical delay activity measured during the delay, especially for trial-to-trial neural variability, and the model-derived markers (memory noise and lapse probability) was also particularly strong in the MCI group. Since a decline in working memory function is a common finding within the MCI diagnosis (Saunders and Summers, 2010), it is nonetheless noteworthy that overt group differences between healthy older subjects and MCI patients are lacking. It is possible that working memory deficits only become apparent in tasks with higher working memory load and/or longer delays, which could be a key difference between our design and previous studies investigating working memory in MCI patients (e.g., with the CANTAB test battery; Saunders and Summers, 2010). In any case, taken together these observations highlight the translational potential of model- and MEG-based assessments of working memory mechanisms for the objective detection and monitoring of cognitive impairment during aging.

Notably, during the working memory delay, MCI patients’ fixational eye movements were more systematically related to the sample stimulus locations than both younger and OHC groups. The higher precision of sample location decoding from gaze direction in the MCI group compared with the other groups could simply be due to relatively larger-amplitude gaze shifts toward the sample locations in the MCI group, possibly due to a reduced ability to suppress fixational eye movements in order to follow the task instruction to maintain steady fixation throughout the trial. Alternatively, the result could reflect a compensatory strategy deployed by the MCI group to ameliorate what would otherwise manifest as clearer performance decrements. That being said, the behavioral relevance of this potential compensatory strategy remains unclear since we found no correlation between the precision of the sample location decoding from gaze direction and task performance.

Our MEG approach builds on recent neuroimaging work that illuminated the possibility of decoding the content and precision of stimulus-selective working memory representations from noninvasively measured spatial patterns of brain activity. Previous functional MRI studies have decoded stimulus-specific information from activity patterns in the frontal, parietal, and visual cortex during working memory delays (Christophel et al., 2017; Curtis and Sprague, 2021). While similar decoding approaches applied to event-related potential signals failed to identify lasting stimulus-selective activity patterns (Wolff et al., 2015, 2017; Rose et al., 2016), they have succeeded when applied to modulations of band-limited signal power (Foster et al., 2016; Barbosa et al., 2020, 2021), as we did here. Moreover, while the decodability of memoranda from these signals may decrease to some degree over memory delays, this can be explained at least in part by the drift in the underlying memory representations that is thought to be a primary source of error in behavioral working memory reports (Wimmer et al., 2014; Wolff et al., 2020). These characteristics—which we observed in patterns of spectral power modulation in the dorsal visual cortex—are generally consistent with cortical circuit models in which working memory emerges from sustained attractor states that drift over time (Compte, 2000; Murray et al., 2014; Wimmer et al., 2014; Schneegans and Bays, 2018).

Another key prediction of these models is that the persistent activity patterns underlying working memory representations should manifest in strong temporal generalization of the underlying code. While this property has been observed previously for EEG spectral power modulations in young adults for a visuospatial working memory task similar to ours (Barbosa et al., 2020), we only found evidence for limited stability of the code during the working memory delay in older adults. This inconsistency might be explained by several factors: (1) our lower data yield in the older participants, which generally decreases decoding accuracy, as well as our capacity to recover a stable code, (2) the decreased stability of older adult memory representations that we have demonstrated presently through our model-based analysis, which will translate into a less stable code, and (iii) perhaps, a greater reliance of the older adult cohort on “activity-silent” mechanisms for working memory (Wolff et al., 2017), which can complement maintenance through persistent activity (Barbosa et al., 2020; Stein et al., 2021). We note that we are currently left to speculation regarding points (1) and (3), but these would be potentially interesting avenues for future research into aging.

Our results complement other studies linking behavioral performance (Murray et al., 2014) and neural signals (Barbosa et al., 2020, 2021) in working memory tasks to the dynamics of cortical circuit models. These links formalize relationships between neural microcircuit properties (e.g., the ratio of recurrent excitation and inhibition in a circuit, E/I) and patterns of task behavior. Thus, such links open opportunities for understanding behavioral effects of cognitive aging in terms of underlying neural circuit properties. For example, there is indirect evidence for alterations of cortical E/I in Alzheimer's disease (Montez et al., 2009; Maestú et al., 2021). One challenge associated with cortical circuit modeling of measured behavioral changes is that the parameter space of cortical circuit models is high-dimensional, and in some cases different circuit alterations can produce highly similar behavioral effects (Stein et al., 2021). Here, we circumvented this issue by fitting a lower-dimensional model, which was well constrained by the behavioral task while exhibiting straightforward relationships to more detailed circuit models (Murray et al., 2014). Complementing such behavioral modeling with noninvasive measures of E/I (Gao et al., 2017; Pfeffer et al., 2018, 2021; Bruining et al., 2020) and other circuit properties may help to add much-needed constraint in future applications of these neural circuit models to empirical data.
