# Supplementary figures and images for: Linking Cognitive Integrity to Working Memory Dynamics in the Aging Human Brain
Source: J Neurosci. 2024 May 17;44(26):e1883232024. doi: 10.1523/JNEUROSCI.1883-23.2024 (PMC11211717; doi:10.1523/JNEUROSCI.1883-23.2024)

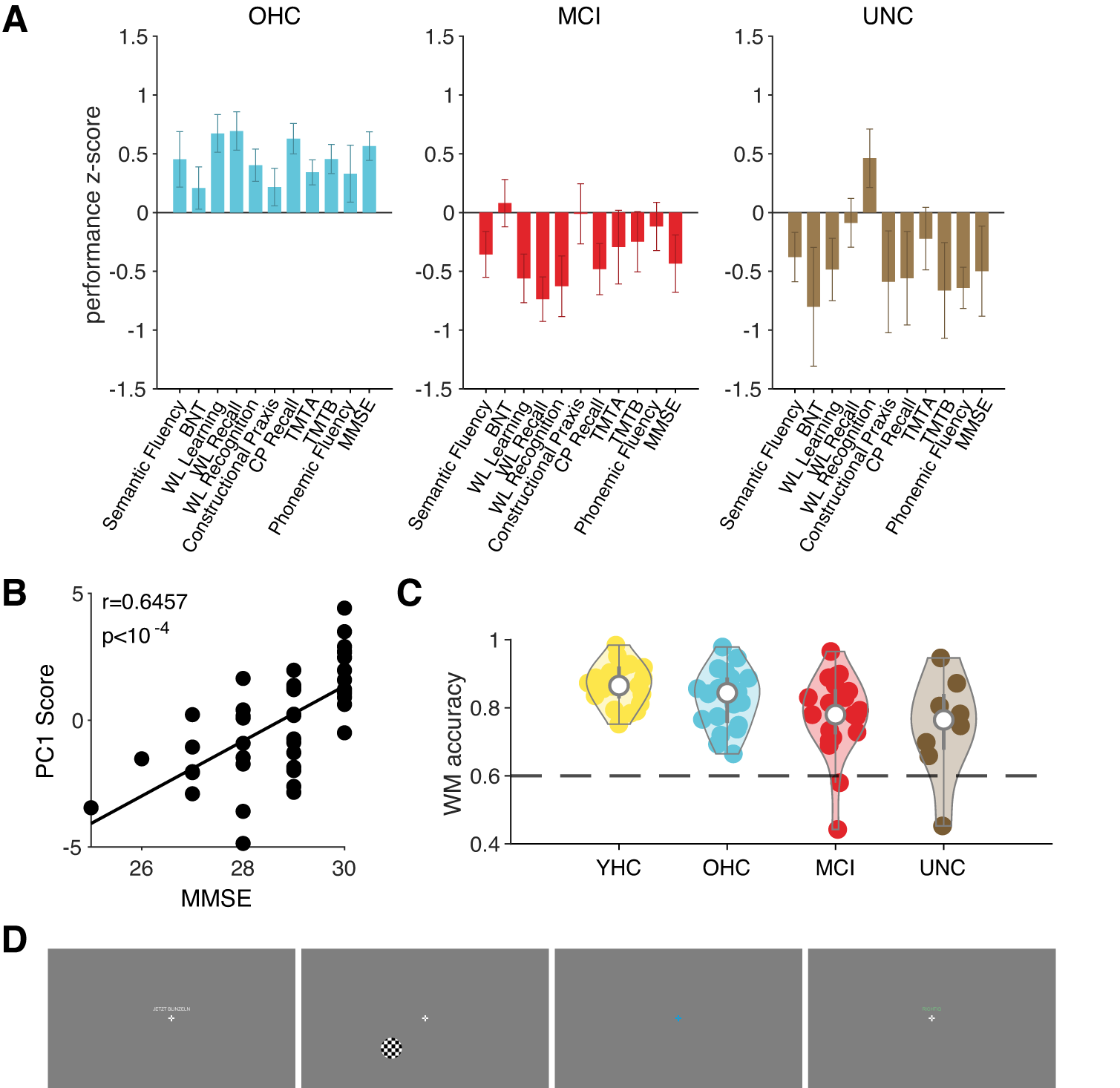

Supplement: Figure 1-1 — CERAD-Plus test battery performance scores, validation of composite cognitive score and performance exclusion criterion. (A) Z-scored CERAD-Plus test results (input to principal component analysis) per group (mean ± s.e.m.) show cognitive performance decline in the MCI and UNC groups compared with the OHC group. (B) PC1 scores and performance on Mini-Mental State Examination (MMSE) are strongly correlated. (C) Single subject working memory task accuracy and kernel density estimates of subgroups show the distributions of task performance measures. The application of task accuracy exclusion criterion at p(correct) = 0.6 (dashed black line) leads to the exclusion of three subjects (MCI, N = 2; UNC, N = 1). (D) True-sized representation of the stimuli shown to the subjects. Left to right: break cue, fixation cross and checkerboard patch, response cue, feedback (correct). Download Figure 1-1, TIF file. [file jneuro-44-e1883232024-s001.tif]

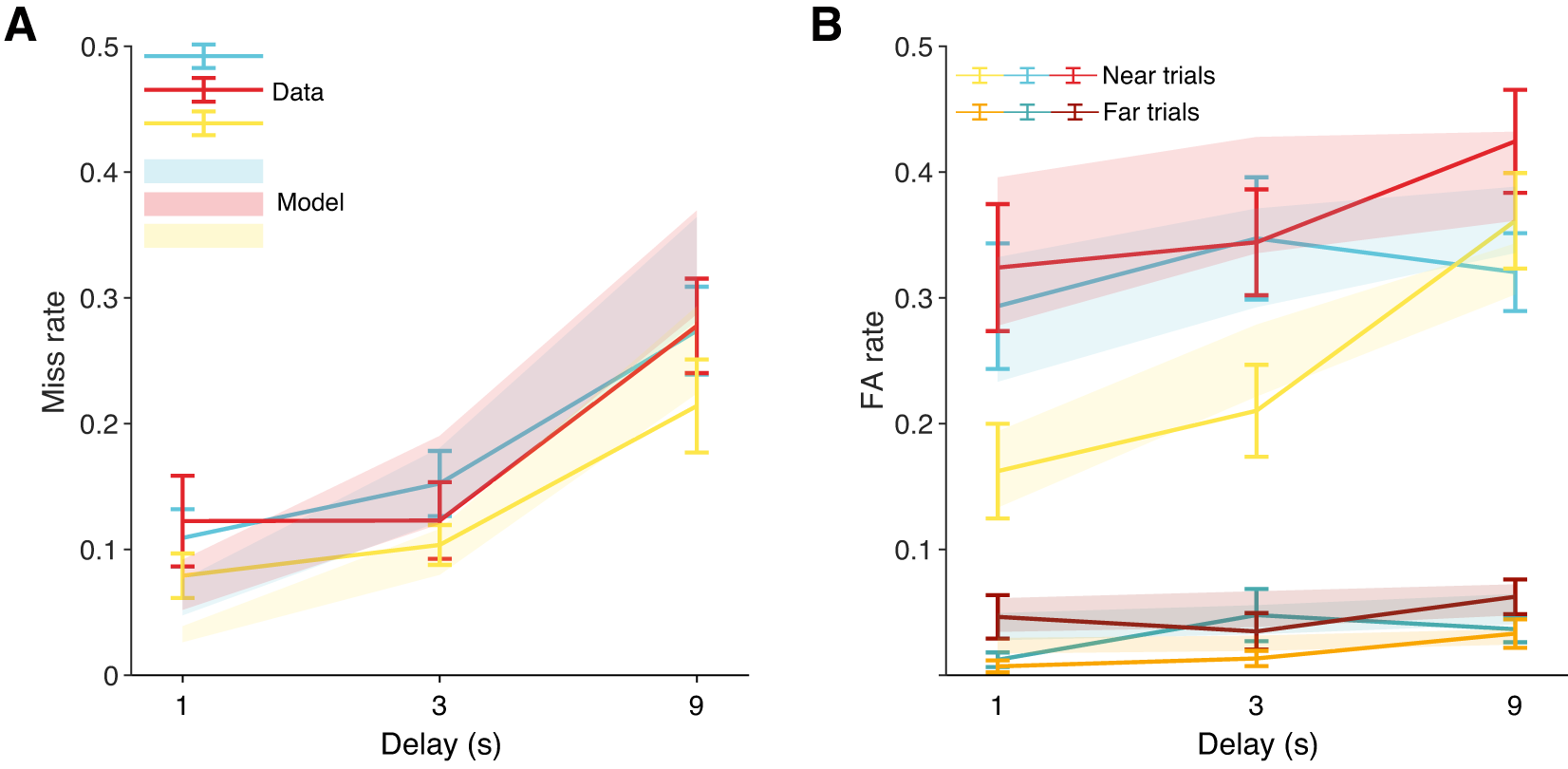

Supplement: Figure 2-1 — Error rates and model predictions on working memory task differentiated by delay duration, sample-test stimulus distance, and participant group. (A) Miss rates (mean ± s.e.m.) by delay duration for YHC (yellow, N = 21), OHC (blue, N = 20) and MCI (red, N = 19). (B) False alarm rates (mean ± s.e.m.) on near non-match trials (lighter shades) and far non-match trials (darker shades). A&B Model predictions (mean ± s.e.m.) are shown as shadings in the corresponding color. Download Figure 2-1, TIF file. [file jneuro-44-e1883232024-s002.tif]

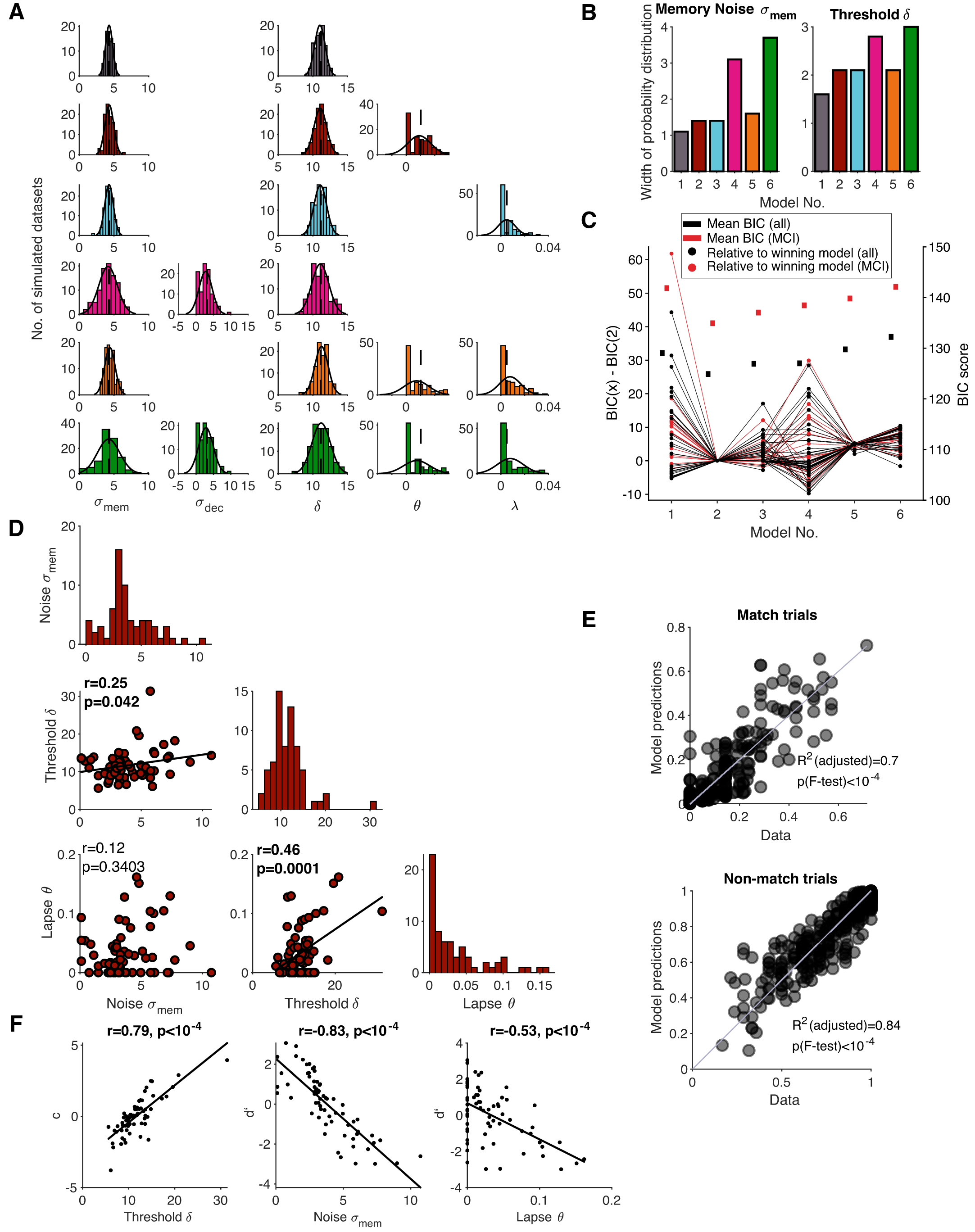

Supplement: Figure 3-1 — Model validation and comparison. (A) Histograms of fitted parameters for simulated data sets (models 1-6, colored), the corresponding fit of a normal density function (solid black line) and parameter levels at which the data sets were simulated (dashed black line). (B) Width of the probability density function for fitted model parameters that are included in all candidate models (memory noise and decision threshold) at half maximum. Models 4 and 6 (including decision noise as a free parameter) showed the strongest limitations in the recoverability of memory noise and threshold. (C) Comparison of the candidate models 1-6 from the mean BIC scores (right y-axis) and the BIC score relative to the winning model (left y-axis) for MCI patients (red) and the entire sample (black), indicating superiority of Model 2. (D) Intercorrelation and histograms of fitted model parameters for Model 2 across all subjects (N = 67). (E) Model predictions of Model 2 were tested for match (top) and non-match trials (bottom) separately. Circles represent predicted probability of a “different” response on 1, 3 and 9 s delay trials per subject (N = 67) as a function of the corresponding observed proportion of behavioral responses for the same trial-type. Non-match trials were segregated into “far” and “near” trials analogous to behavioral analyses. Statistics refer to linear regression model fits of predicted and actual responses. Grey solid lines represent the identity line. (F) Correlations of fitted model parameters (N = 67) with standard SDT measures of criterion (c) and sensitivity (d’) further serve as validation of fitted model parameters. Download Figure 3-1, TIF file. [file jneuro-44-e1883232024-s003.tif]

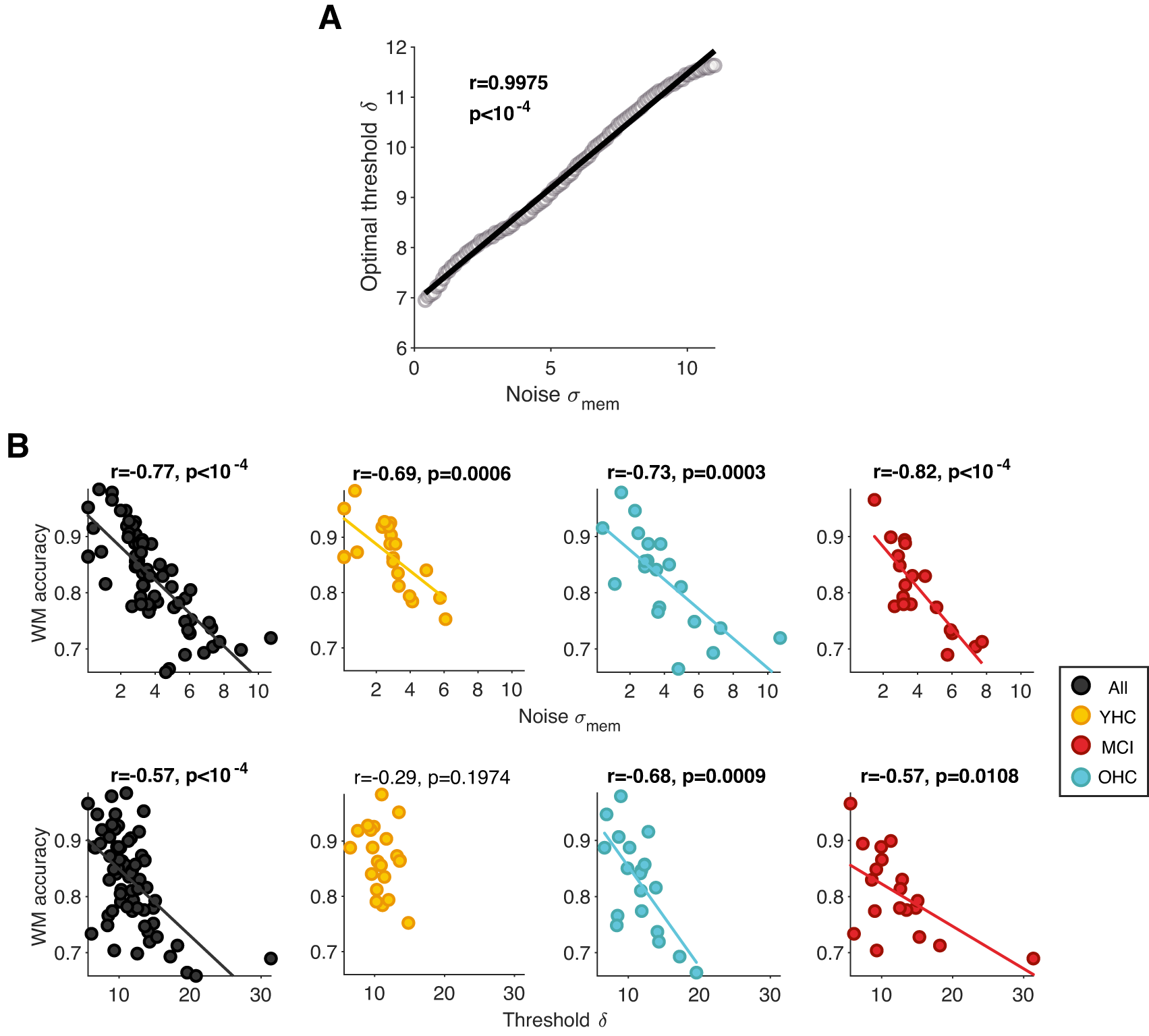

Supplement: Figure 3-2 — Relationship of memory noise and threshold parameters. (A) Memory noise levels and corresponding optimal threshold fits are strongly positively correlated in simulated data showing an optimality interdependence of the two parameters. (B) Correlation of working memory accuracy with fitted noise (top) and threshold (bottom) parameters for all subjects (black, N = 67), YHC (yellow, N = 21) OHC (blue, N = 20) and MCI (red, N = 19). Across all participant groups, high noise parameters are related to decreased accuracy on the working memory task. Such a relationship with threshold parameters is present across the entire sample, however on a subgroup level it can only be found in the older subjects. There is no significant difference in the correlation coefficients between YHC and older subjects or either of the older participant groups separately (two-sided permutation tests on Δr, all p > 0.14). Download Figure 3-2, TIF file. [file jneuro-44-e1883232024-s004.tif]

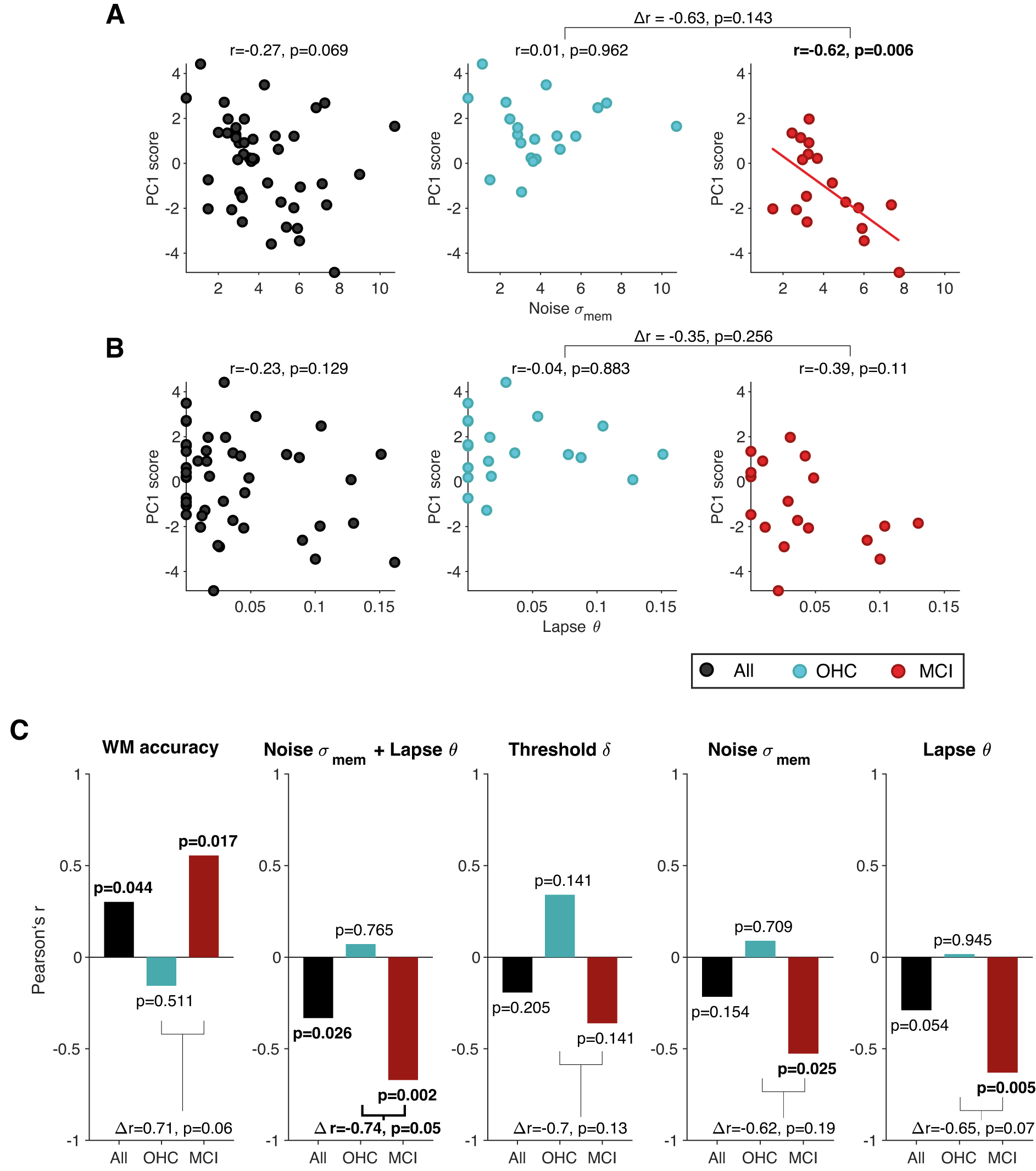

Supplement: Figure 4-1 — Correlation analyses with cognitive integrity scores. Correlation of PC1 scores with (A) noise parameter fits and (B) lapse parameter fits. In all panels, circles represent individual subjects within the group of older participants. Correlations and correlational statistics are reported for all older subjects (N = 45, black, left), OHC (N = 20, blue, middle) and MCI (N = 18, red, right) separately. Linear regression fit is shown for statistically significant correlations only. Differences in correlations between OHC and MCI are shown on top of the square brackets, p-values refer to two-sided permutation tests. (C) Correlational analysis for behavioral measures derived from the WM task with established CERAD total scores (analogous to analysis with PC1 scores). Bar graphs depict Pearson’s correlation coefficient with the corresponding p-value on top of each bar. Results are expectedly similar to the same correlations with PC1 scores (Fig. 4). Download Figure 4-1, TIF file. [file jneuro-44-e1883232024-s005.tif]

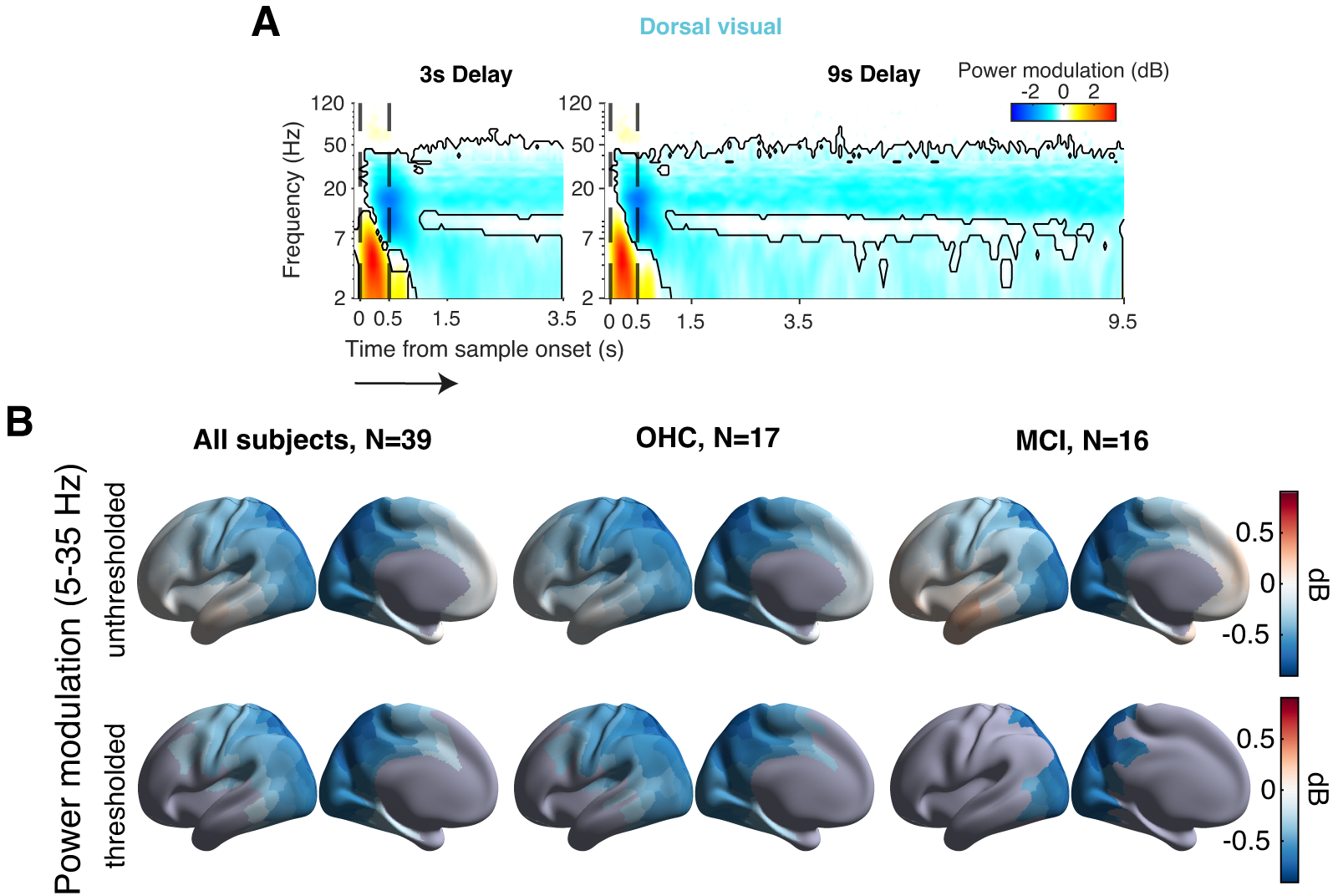

Supplement: Figure 5-1 — Power modulation on longer delay trials and brain maps for fine-grained cortical parcellation. (A) Task-induced power modulations for dorsal visual clustered brain region (see Table 3) for 3 s delay (including first three seconds of trials with a 9 s delay duration) and 9 s delay show the persistence of power suppression with relative omission of alpha frequencies beyond the first second of delay (all subjects, N = 39). Black contouring refers to significant within-subjects power modulation (cluster-based permutation test, p < 0.05). (B) Fine-grained parcellated brain maps (N = 180 ROIs) of unthresholded (top) and FDR-corrected (bottom) low-frequency (5-35 Hz) power modulation during first second into delay duration (significance threshold, p < 0.05). Download Figure 5-1, TIF file. [file jneuro-44-e1883232024-s006.tif]

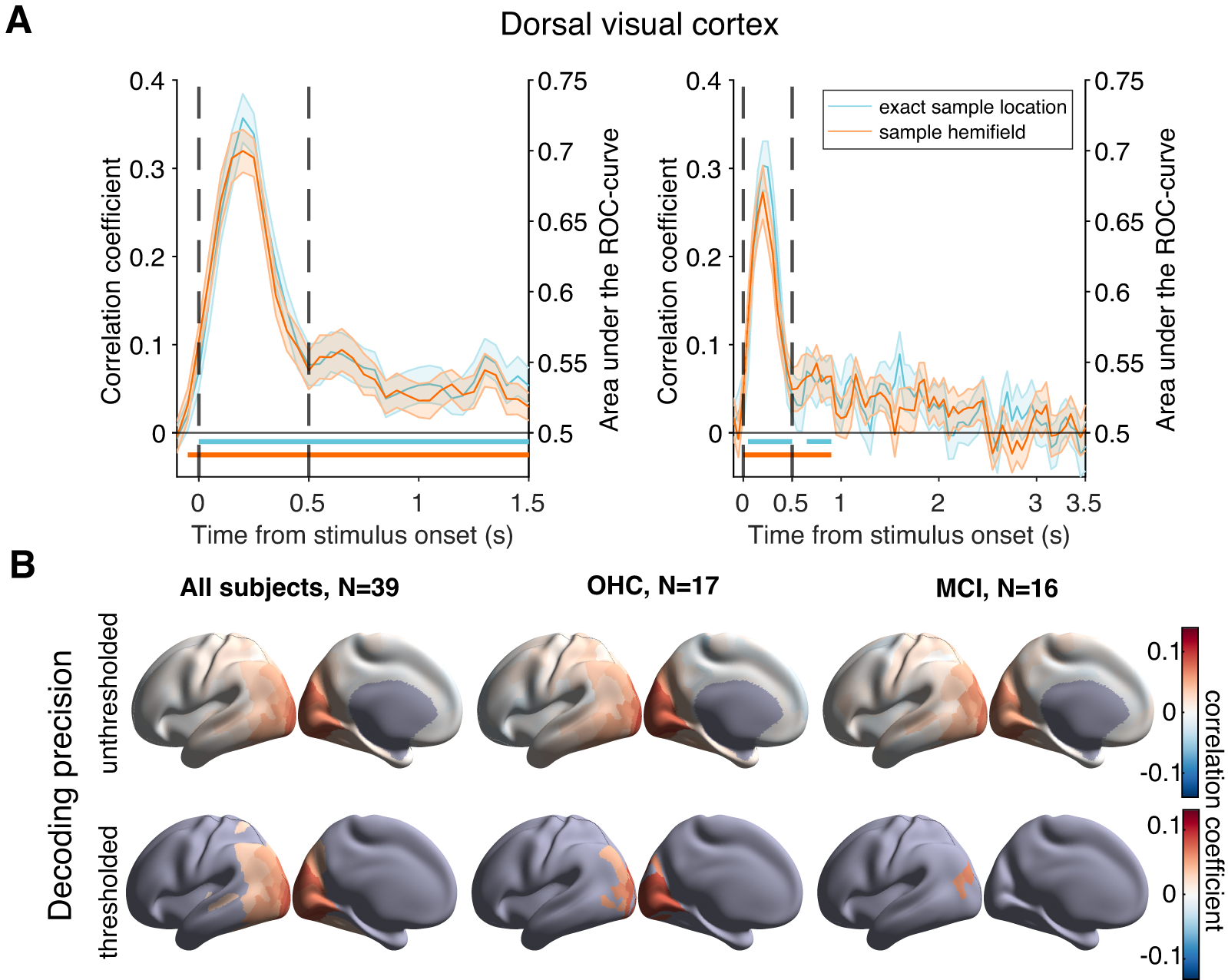

Supplement: Figure 6-1 — Decoding precision of hemifield and exact sample location on longer delay trials and brain maps for fine-grained cortical parcellation. (A) Encoding of sample hemifield (orange) vs. exact sample location (blue) in dorsal visual cortex. Time courses of vertex-based decoding precision (power values from 5-35 Hz; all older subjects, N = 39; mean ± s.e.m.) for encoding of the exact sample location (correlation coefficient of predicted and actual location; left y-axis) and the sample hemifield (area under the ROC curve from prediction scores for classification of hemifield; right y-axis) for 1 s delay duration (left) and 3 s delay duration (right). Dashed lines represent sample stimulus onset and offset. Solid horizontal lines in the corresponding color represent latencies of significant decoding precision (cluster-based one-sided permutation test against zero for correlation coefficients or against 0.5 for AUC, p < 0.05). Although dorsal visual cortex showed robust encoding when considering only the first second of the delay period of all trials, no significant decoding precision could be demonstrated throughout the 3 s delay duration. Decoding of the exact location and the sample hemifield yield comparable results. (B) Fine-grained parcellated brain maps (N = 180 parcels) of unthresholded (top) and FDR-corrected (bottom) decoding precision during first second into delay duration (significance threshold, p < 0.05). Download Figure 6-1, TIF file. [file jneuro-44-e1883232024-s007.tif]

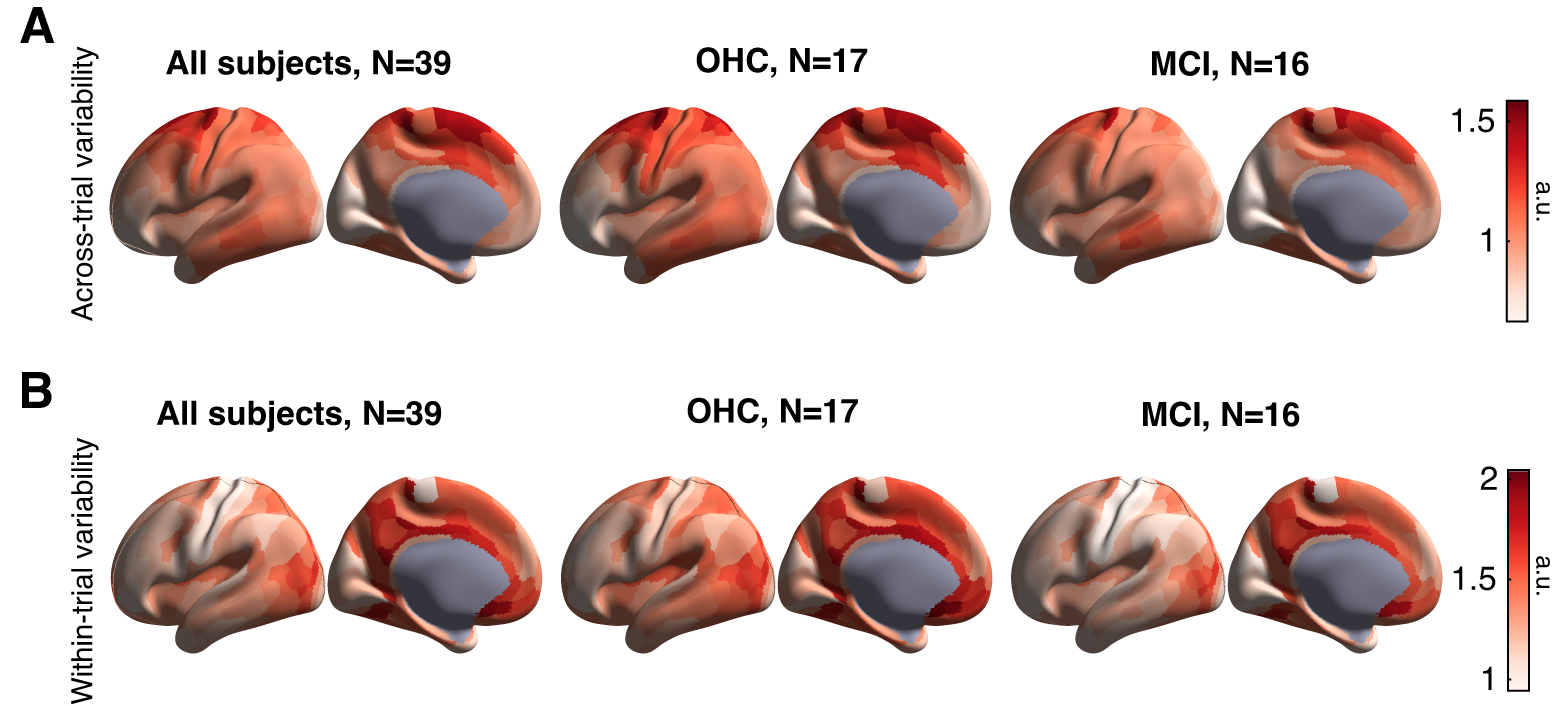

Supplement: Figure 7-1 — Cortical distributions of variability of task-related MEG power. (A) Maps of across-trial variability in power modulations during 1 s of delay duration in the frequency range of 5-35 Hz. (B) As A, but for within-trial variability in power modulations. Download Figure 7-1, TIF file. [file jneuro-44-e1883232024-s008.tif]

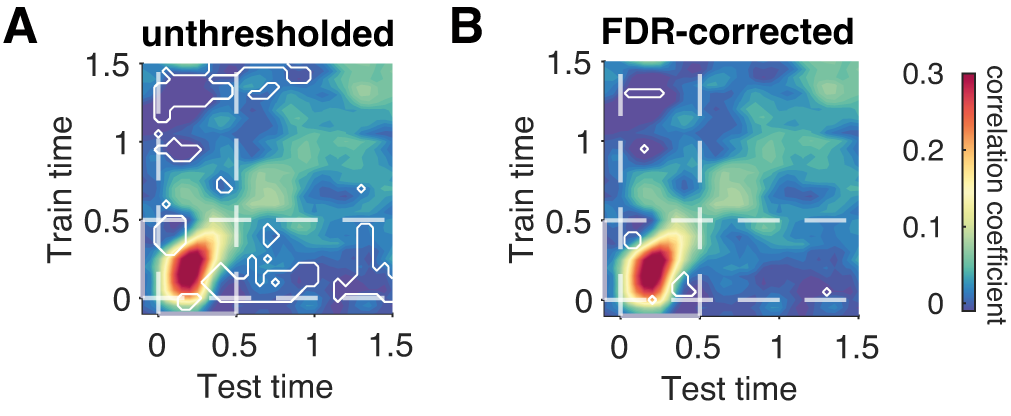

Supplement: Figure 8-1 — Testing dynamic working memory code. Averaged temporal generalization matrices of all older subjects (N = 39) in dorsal visual cortex. Dashed white lines depict sample stimulus onset and offset. White contouring captures time points of dynamic coding (significance threshold, p < 0.05, (A): unthresholded; (B): after FDR-correction). Download Figure 8-1, TIF file. [file jneuro-44-e1883232024-s009.tif]

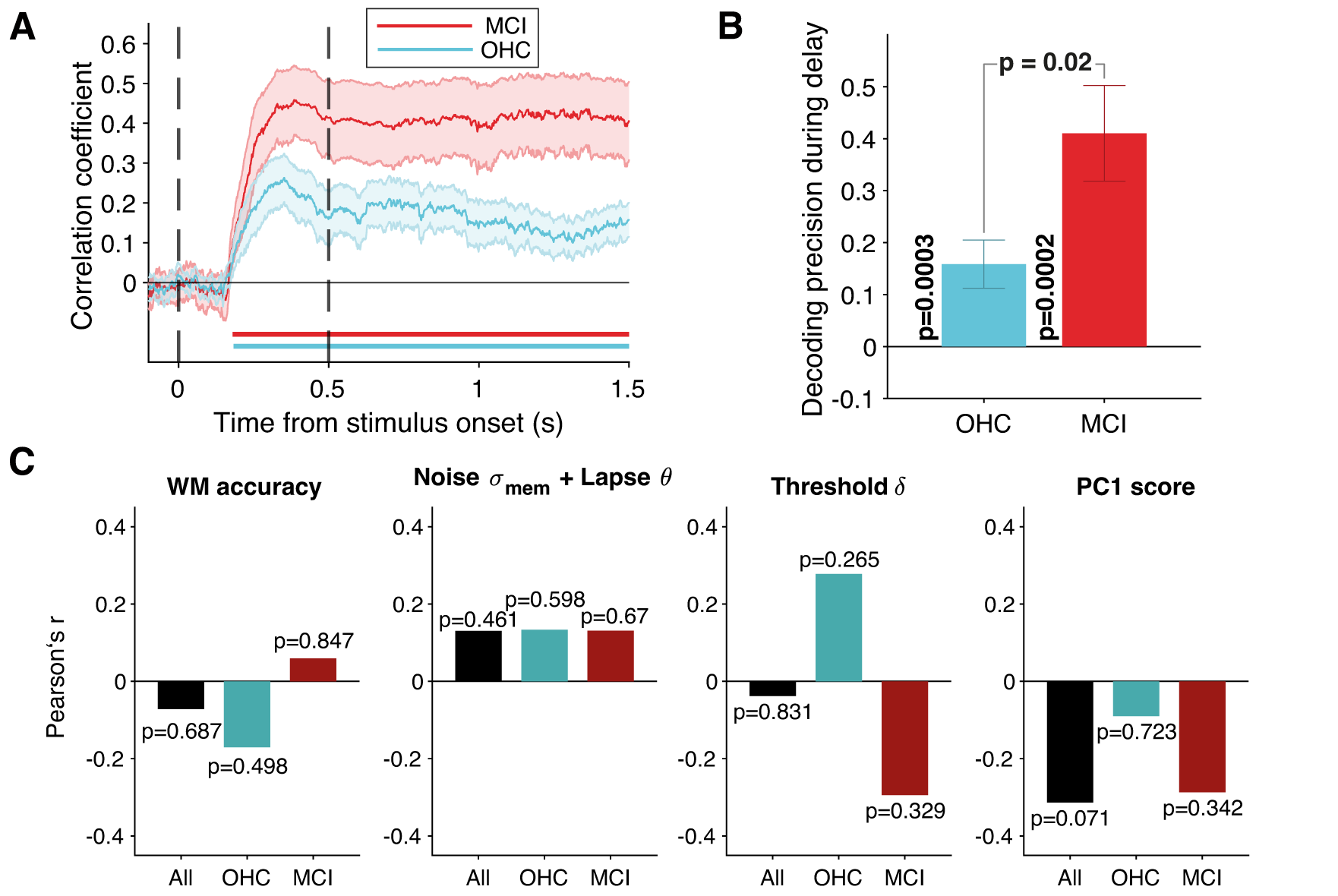

Supplement: Figure 9-1 — Analysis of gaze directions at higher temporal resolution (1000 Hz) for older subjects. (A) Time courses of decoding precision from gaze direction data for MCI (red) and OHC (blue) groups separately. Dashed lines depict sample stimulus onset and offset. Solid horizontal lines in the corresponding color represent latencies of significant decoding precision (cluster-based one-sided permutation test against zero, p < 0.05). (B) Mean decoding precision during the delay period (1 s) for OHC (N = 18, blue) and MCI (N = 13, red). P-values on the side of each bar graph correspond to within-subjects non-parametric permutation tests against zero (one-sided). (C) Correlation coefficients of mean decoding precision with behavioral measures derived from the working memory task and PC1 scores with corresponding p-values on top of each bar graph for all subjects (N = 34, black), OHC (N = 18, blue) and MCI (N = 13, red). Download Figure 9-1, TIF file. [file jneuro-44-e1883232024-s010.tif]
