# Supplementary material for: Linking Cognitive Integrity to Working Memory Dynamics in the Aging Human Brain
Source: J Neurosci. 2024 May 17;44(26):e1883232024. doi: 10.1523/JNEUROSCI.1883-23.2024 (PMC11211717; doi:10.1523/JNEUROSCI.1883-23.2024)
Supplement: Table 1-1 — Group comparison MCI vs. OHC for CERAD-Plus subtest performance. Download Table 1-1, DOCX file. [file jneuro-44-e1883232024-s011.docx]

**Extended Data Table 1-1. Group comparison MCI vs. OHC for CERAD-Plus subtest performance.**

| **Subtest** | **MCI**  mean (S.D.) | **OHC**  mean (S.D.) | **p** |
| --- | --- | --- | --- |
| Word list learning | 17.2 (4.1) | 22.9 (3.4) | **<10^-4^** |
| Word list delayed recall | 4.8 (1.9) | 8.1 (1.7) | **<10^-4^** |
| Discriminability | 92.8 (6) | 98.3 (3.4) | **0.0013** |
| Boston naming test | 14.6 (0.6) | 14.7 (0.6) | 0.7837 |
| Semantic fluency | 20.4 (6) | 26.2 (7.6) | **0.0157** |
| Constructional praxis | 10.2 (1.2) | 10.4 (0.8) | 0.5075 |
| Constructional praxis recall | 7.1 (2.4) | 9.9 (1.5) | **<10^-4^** |
| TMTA | 51.1 (26.5) | 38.8 (9.4) | 0.0521 |
| TMTB | 125.7 (62.4) | 86.5 (31.7) | **0.0127** |
| Phonemic fluency | 13.8 (4.1) | 15.9 (5.1) | 0.1845 |
